# An Efficient Synthesis of Pyridoxal Oxime Derivatives under Microwave Irradiation

**DOI:** 10.3390/molecules19067610

**Published:** 2014-06-06

**Authors:** Dajana Gašo-Sokač, Valentina Bušić, Mario Cetina, Marijana Jukić

**Affiliations:** 1 Josip Juraj Strossmayer University of Osijek, Faculty of Food Technology Osijek, Kuhačeva 20, 31000 Osijek, Croatia; E-Mail: valentina.busic@ptfos.hr; 2 University of Zagreb, Faculty of Textile Technology, Department of Applied Chemistry, Prilaz baruna Filipovića 28a, 10000 Zagreb, Croatia; 3 Faculty of Food Technology and Biotechnology, Department of Chemistry and Biochemistry, Pierottijeva 6, 10000 Zagreb, Croatia; E-Mail: mjukic@pbf.hr

**Keywords:** eco-friendly quaternization, microwave synthesis, phenacyl bromides, pyridoxal oxime

## Abstract

Quaternary salts of pyridoxal oxime have been synthesized by the quaternization of pyridoxal oxime with substituted phenacyl bromides using microwave heating. Microwave-assisted rapid synthesis was done both in solvent (acetone) and under solvent-free conditions. Good to excellent yields (58%–94%) were obtained in acetone in very short reaction times (3–5 min) as well as in the solvent-free procedure (42%–78%) in very short reaction times (7–10 min) too. Effective metodologies for the preparation of pyridoxal oxime quaternary salts, having the advantagies of being eco-friendly, easy to handle, and performed in shorter reactions time are presented. The structure of compound **7**, in which a 4-fluorophenacyl moiety is bonded to the pyridinium ring nitrogen atom, was unequivocally confirmed by the single-crystal X-ray diffraction method.

## 1. Introduction

The development of cleaner technologies is a major emphasis in green chemistry. Among the several aspects of green chemistry, avoiding the use of volatile organic solvents in the reaction medium is recommended. The use of the large excesses of conventional volatile solvents required to conduct a chemical reaction creates ecological and economical concerns. Therefore the search for a nonvolatile and recyclable alternative, as well as performing reactions without solvents has a key role in this field of research [1].

During recent years, microwaves (MW) have been extensively used for carrying out chemical reactions and have become a useful non-conventional energy source for performing organic synthesis. The application of microwaves in chemistry is extremely attractive and, from the very beginning it was realized that a number of chemical processes can be carried out with substantial reduction of the reaction time in comparison to conventional methods [2].

Several papers have been reported on the alkylation of *N*-containing heterocycles [3,4,5,6]. The first solventless quaternization via microwave heating has been described for preparation of variety of ionic liquids [1]. Perez and coworkers have described the *N*-alkylation of azoles with 4-bromophenacyl bromide under MW irradiation under solvent-free conditions. The results obtained showed high yields and selectivity [7].

Organophosphorus compounds are widely used in agriculture as insecticides, in industry and technology, as well as in military technology as chemical warfare agents (sarin, soman, tabun). They are extremely potent inhibitors of the enzyme acetylcholinesterase (AChE) that is responsible for the termination of the action of acetylcholine at cholinergic synapses [8,9]. There are many commonly used reactivators of inhibited AChE, such as 2-pralidoxime, trimedoxime, and toxogonin [10,11,12]. Unfortunately, none of the currently used oximes is sufficiently effective against all inhibitors and there is no single reactivator having the ability to reactivate inhibited enzyme, regardless of the inhibitor chemical structure [13,14].

Previously, we synthesized a series of novel pyridinium oximes and tested them as reactivators of AChE inhibited by organophosphosphorus compounds tabun and paraoxon [15]. In view of the emerging importance of the quartenary salts as antidotes, antibacterial and anticancerogenic agents and our general interest in microwave-assisted chemical processes, we decided to explore the synthesis of those compounds using MW irradiation in the presence of solvents and under solvent-free conditions. In our earlier paper [15] we prepared a series of novel pyridinium oximes under classical conditions. These reactions have some major disadvantages, including long reaction times (1–3 weeks), the usage of large amounts of solvents, *etc.* For these reasons we decided to use MW technology, as a nonconventional method for their synthesis. Herein, we report an efficient method for the preparation of quaternary salts that simply involves exposing neat reactants to MW using a Milestone single-mode microwave reactor.

## 2. Results and Discussion

**Scheme 1:**
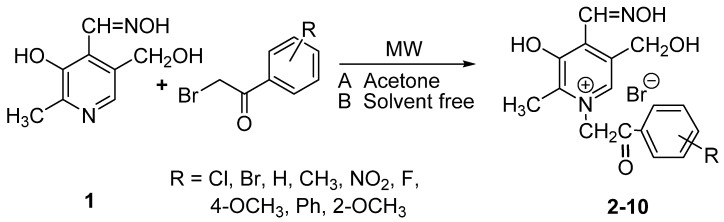
A general schematic representation for the preparation of quaternary salts of pyridoxal oxime.

In this article two MW techniques are presented: MW irradiation in the presence of solvent (acetone) and MW irradiation under solvent free-conditions. The work up was easy and the products were obtained in excellent to moderate yields (as shown in Table 1). This method is efficient, versatile and generates little waste. The results clearly demonstrate quaternization by the classical method is possible as well as via the MW irradiation method, but the synthetic efficiency was significantly different, as evidenced by the data of Table 1. Scheme 1 outlines the synthesis of **2**–**10**. Optimum conditions for carrying out the MW-assisted quaternizations were ascertained by carrying out a series of reactions of pyridoxal oxime with substituted phenacyl bromides. The results showed that maximum yield of 94% was obtained by microwave-assisted procedure in acetone for **5**, while maximum yield of 79% was obtained in MW solvent-free procedure for **6**. All the products were crystalline crude products.

**Table 1:** The optimized conditions in the synthesis of quaternary salts under MW heating in acetone A and solvent-free procedure B as well as under classical heating.

Compound	R	MW (A)		MW (B)		Conventional [15]	
		t/min	Yield (%)	t/min	Yield (%)	t/weeks	Yield (%)
2	4'-Cl	5	75	10	63	3	66
3	4'-Br	4.5	90	10	74	3	44
4	4'-H	5	74	7	53	1	38
5	4'-CH_3_	4	94	8	76	1	62
6	4'-NO_2_	4.5	80	10	79	1	60
7	4'-F	3	70	10	48	3	36
8	4'-OCH_3_	5	90	10	71	3	67
9	4'-Ph	5	79	10	46	1	46
10	2'-OCH_3_	5	58	10	42	12	12

Interestingly, the same reactions carried out under conventional conditions using acetone, methanol and DMF as a solvents gave **2**–**10** in lower yields, and required significantly longer reaction time [15].

The syntheses were done in a Milestone controllable single-mode microwave reactor. The reactor is equipped with a magnetic stirrer as well as temperature and power controls. The effect of microwave irradiation on a set of reactions using pyridoxal oxime and substituted phenacyl bromides as reactants was examined. Under these conditions a very efficient, fast, and practical method for the preparation of quaternary salts was developed. The time required to synthesize the salts compared to conventional method is strongly reduced.

In case of the microwave-assisted reactions using organic volatile solvents, the reactants are usually dissolved in the solvent, which often couples effectively with microwaves and thus acts as the energy transfer medium. Acetone was selected as MW solvent because the highest yield has been obtained with it compared to those obtained in DMF and methanol via the conventional technique [15]. The reaction carried out in acetone via MW irradiation required a maximum of 5 min of irradiation. The white and light yellow color of the reactants turned yellow brownish as the mixing and irradiation progressed.

In the microwave-assisted quaternizations in acetone the products required no rigorous purification and pure products were obtained by simple recrystallization from an appropriate solvent. Therefore, this method offers an easy practical access for the production of a series of quaternary salts.

Solvent-free methods are especially adapted to organic synthesis under green chemistry conditions. When coupled to MW irradiation, these methods result in very efficient and noticeable improvements over classical methods. The absence of solvent reduces the risk of explosions, moreover, aprotic solvents with high boiling points are expensive and difficult to remove from the reaction mixtures. This solvent-free approach requires only a few min of reaction time in contrast to few weeks under conventional heating conditions. Although this synthesis offers several advantages over traditional methods, there is still need for improving the postsynthetic treatment where solvents are also used. Quaternization of pyridoxal oxime with substituted phenacyl bromides was accelerated under MW irradiation. Adsorption of reactant molecules on the surface of silica gel was promoter force for this reaction. It was interesting that without silica gel added the reaction was not successful. The silica gel surface was an active catalyst for reaction of quaternization in this solventless procedure. In spite of the fact that “dry” microwave procedure demand easy work up it is obvious that purification in procedure done in acetone is increasingly shorter and less rigorous. The solventless quaternization MW procedure requires only a few min of reaction time but purification was quite longer as compared with MW procedure in acetone due to the presence of starting materials and some byproducts.

The lowest yields in both the solvent-free method (42%) and in acetone (58%) was obtained for the quaternization of pyridoxal oxime with 2-bromo-1-(4-metoxyphenyl)ethanone. This low yield can be explained by the existence of an *ortho*-methoxy substituent in the electrophile which prevents attack on the pyridoxal oxime at the nitrogen atom due to steric hindrance.

**Figure 1:**
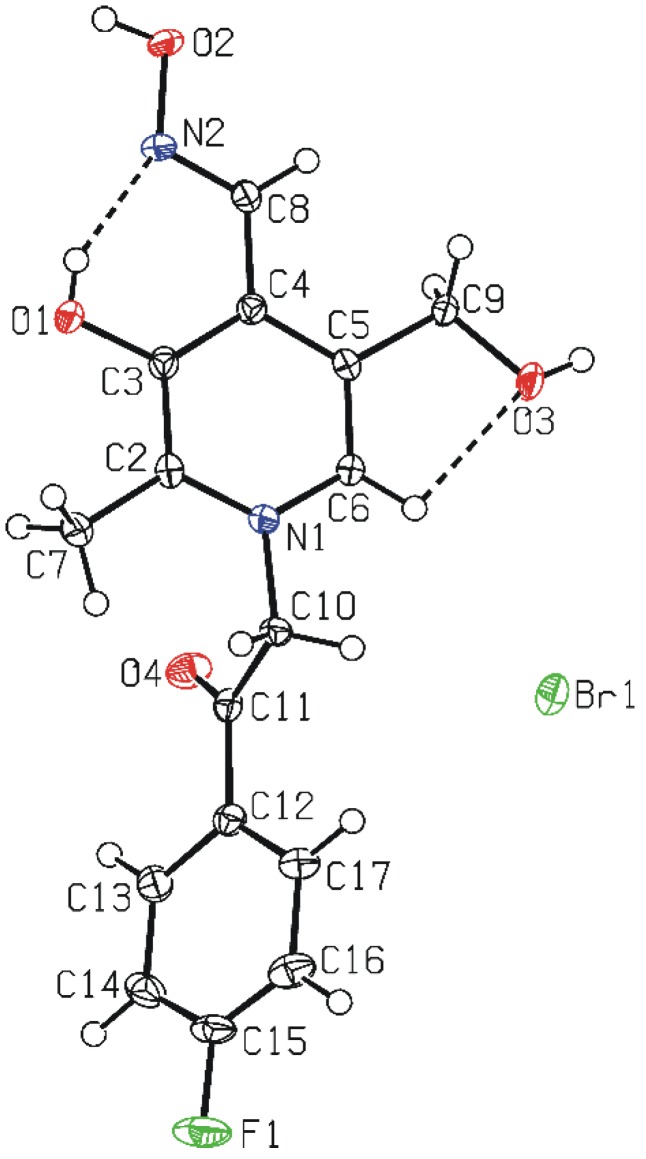
A molecular structure of **7**, with the atom-numbering scheme. Displacement ellipsoids for non-hydrogen atoms are drawn at the 50% probability level.

The structure of compound **7** was unequivocally confirmed by single-crystal X-ray diffraction method. In **7** (Figure 1), the 4-fluorophenacyl moiety is bonded to the pyridinium ring N1 atom. The C2-N1-C6 bond angle in the pyridinium ring is widened [123.2(2)°]. However, sum of the endocyclic bond angles is 720°, as expected for aromatic six-membered ring. The bond lengths and angles agree quite closely with equivalent ones in structures of 3-hydroxy-4-hydroxyiminomethyl-5-hydroxymethylpyridinium derivatives [16,17]. The C2 ring atom is in *synclinal* position with respect to the C11 atom of the phenacyl moiety [C2−N1−C10−C11 = 77.2(3)°]. This indicates that phenyl ring is rotated with respect to the pyridinium ring, as also shown by the dihedral angle between mean ring planes of 55.38(13)°.

In the cation, one strong O−H···N intramolecular hydrogen bond [O1···N2 = 2.579(3) Å] forms six-membered ring, and one C−H···O intramolecular hydrogen bond [C6···O3 = 2.648(3) Å] generates five-membered ring (Figure 1).

**Figure 2:**
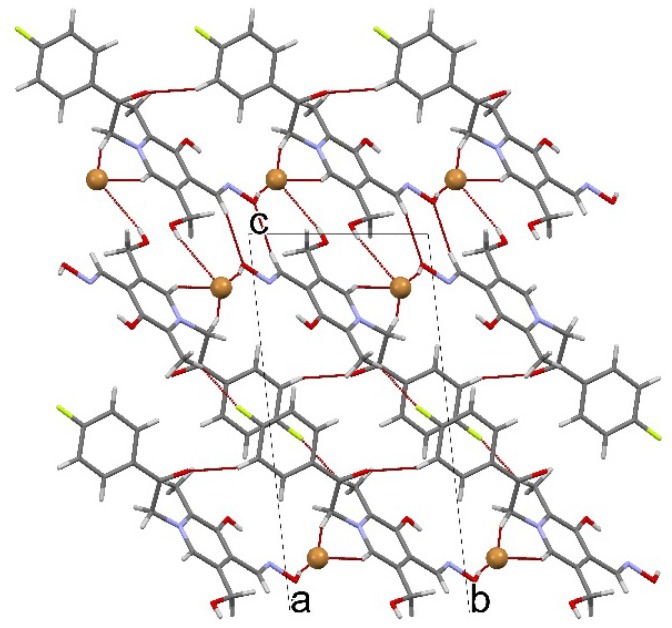
Crystal packing diagram along the *a* axis, showing three-dimensional network formed by O−H···Br^−^, C−H···Br^−^, C−H···O and C−H···F intermolecular hydrogen bonds. Bromides are presented in ball and stick style.

The pyridinium cation and bromide are linked by various intermolecular interactions. Two pyridinium cations and two bromides are assembled by one strong O−H···Br^−^ hydrogen bond [O3···Br1 = 3.451(2) Å] and two C−H···Br^−^ hydrogen bonds [C10···Br1 = 3.667(3) Å; C6···Br1 = 3.817(3) Å], so forming ring consists of two cations and two anions (Figure 2).

This ring is further linked by O−H···Br^−^, C−H···O i C−H···F hydrogen bonds [O2···Br1 = 3.131(2) Å; C8···O2 = 3.479(3) Å; C16···O4 = 3.486(4) Å; C7···F1 = 3.136(4) Å] into three-dimensional network. One π...π interaction participates also in supramolecular assembling. The phenyl rings of the neighbouring molecules are mutually parallel [*α* = 0°], a centroid separation is 3.6374(17) Å, an interplanar spacing *ca* 3.33 Å and offset *ca.* 1.46 Å.

## 3. Experimental Section

### 3.1. General Information

All reactions were performed using a, Microwave Synthesis Labstation Start S controllable single-mode microwave reactor (Milestone, Shelton, CT, USA). The reactor is equipped with a magnetic stirrer as well as a temperature and power controls (220 V/50–60 Hz, 2.4 kW). Solvents and reagents were purchased from Fluka (Milwaukee, WI, US) and Aldrich (St. Luis, MO, US) and used without further purification. TLC was done by using chloroform–methanol (6:1.5, v/v) as the developing solvent. The silica gel (Aldrich, 0.063–0.200 nm; 70–230 mesh) was used in solvent-free method. IR spectra were measured on a FTIR-8400S spectrophotometer (Shimadzu, Kyoto, Japan) in KBr pellets. ^1^H-NMR and ^13^C-NMR spectra were measured on a XL-GEM 300 spectrophotometer (Varian, Rheinstetten, Germany) in DMSO-*d*_6_ solutions and chemical shifts are reported in δ values downfield from TMS as an internal standard. The compounds are also characterized by elemental analyses. Melting points were determined with a SMP3 melting point apparatus (Stuart, Staffordshire, UK).

### 3.2. General Procedure for the Synthesis of Compounds **2**–**10** with MW Procedure A

Pyridoxal oxime (0.18 g; 1 mmol) was dissolved in acetone (50 mL) at 50 °C. The reaction mixture was cooled to room temperature, and substituted phenacyl bromide was added (1 mmol). The mixture was placed in the Milestone single-mode microwave reactor and irradiated at with starting power at 440 W and reaction temperature 56 °C until TLC analysis has shown the presence of the product. When the irradiation was stopped the mixture was left in the dark to cool and the crystalline crude product was collected by filtration under reduce pressure and recrystallized from appropriate solvent.

### 3.3. General Procedure for the Solvent-Free Synthesis of Compounds **2**–**10** with MW Procedure B

Pyridoxal oxime (0.18 g; 1 mmol) and substituted phenacyl bromide (1 mmol) was added into the mortar and the mixture was grinded with pestle in the presence of silica gel (500 mg) for 10 min. The mixture was placed in the microwave reactor and irradiated with a starting power at 440 W and reaction temperature 70 °C until TLC has shown the presence of the product. Reaction times are reported in the Table 1. The mixture was dissolved in hot acetone. Silica gel was removed from the mixture by filtration under reduced pressure and residual crude was collected by filtration under reduced pressure and recrystallized from appropriate solvent. All products were analyzed by ^1^H- and ^13^C-NMR, IR spectroscopy and elemental analyses.

### 3.4. Characterization Data

*1-(4-Chlorophenacyl)-3-hydroxy-4-hydroxyiminomethyl-5-hydroxymethyl-2-methylpyridinium bromide* (**2**). Yellow solid; R_f_ value: 0.18 (chloroform‒methanol, 6:1.5, v/v); mp after crystallization from methanol 230–233 °C; FT-IR (KBr) *ν*_max_: 3385, 3073–2858, 1688, 1637–1589, 1261, 1051–1003 cm^−1^; ^1^H-NMR: *δ* 12.78 (1H, bs, NOH), 8.67 (1H, bs, OH), 8.59 (1H, s, H-6), 8.14–8.11 (2H, d, *J* = 8.62 Hz, H-2', H-6'), 7.77–7.74 (2H, d, *J* = 8.58 Hz, H-3', H-5'), 6.89 (2H, s, CH_2_CO), 5.84 (1H, bs, CH_2_OH), 4.81 (2H, s, CH_2_OH), 2.51 (3H, s, CH_3_); ^13^C-NMR: *δ* 189.82 (C, C=O), 152.55 (C, C-3), 145.46 (C, C-2), 139.65 (C, C-6), 137.28 (C, C-4), 135.10 (C, C-4'), 132.25 (C, C-1'), 130.43 (C-2', C-6'), 129.76 (C-3', C-5'), 128.98 (C, C-5), 64.48 (CH_2_CO), 58.47 (CH_2_OH), 13.32 (CH_3_); MS (*m/z*): 415.0 [M]^+^ (54.16), 335.2 (43.05, Br), 333.0 (100), 302.8 (39.58), 164.1 (36.11); Anal. Calcd. for C_16_H_16_N_2_O_4_BrCl: C 46.23, H 3.88, N 6.74; Found: C 46.34, H 3.86, N 6.72%.

*1-(4'-Bromophenacyl)-3-hydroxy-4-hydroxyiminomethyl-5-hydroxymethyl-2-methylpyridinium bromide* (**3**) Yellow solid; R_f_ value: 0.27 (chloroform‒methanol, 6:1.5, v/v); mp after crystallization from ethyl acetate 195–198 °C; FT-IR (KBr) *ν*_max_: 3385, 3073-2849, 1686, 1636–1456, 1261, 1049–980 cm^−1^; ^1^H-NMR: *δ* 12.99 (1H, bs, NOH), 8.66 (1H, bs, OH), 8.63 (1H, s, H-6), 8.04-8.01 (2H, d, *J* = 8,61 Hz, H-2', H-6'), 7.92–7.89 (2H, d, *J* = 8.55 Hz, H-3', H-5'), 6.61 (2H, s, CH_2_CO), 5.88 (2H, s, CH_2_OH), 4.81 (1H, s, CH_2_OH), 2.3 (3H, s, CH_3_); ^13^C-NMR: *δ* 190.02 (C, C=O), 152.47 (C, C-3), 145.44 (C, C-2), 137.28 (C, C-6), 135.11 (C, C-4), 132.56 (C, C-4'), 132.06 (C, C-1'), 130.43 (C-2', C-6'), 128.93 (C-3', C-5'), 128.16 (C, C-5), 64.37 (CH_2_CO), 58.46 (CH_2_OH), 13.28 (CH_3_); MS (*m/z*): 460.8 [M]^+^ (17.48), 379.1 (100), 361.1 (39.16), 349.1 (39,86), 199.0 (34.96), 160.7 (39.16); Anal. Calcd. for C_16_H_16_N_2_O_4_Br_2 _: C 41.77; H 3.50; N 6.09; Found: C 41.96, H 3.61, N 5.97%.

3*-Hydroxy-4-hydroxyiminomethyl-5-hydroxymethyl-2-methyl-1-phenacylpyridinium bromide* (**4**). Yellow solid; R_f_ value: 0.26 (chloroform‒methanol, 6:1.5, v/v); mp after crystallization from methanol 250–251 °C; FT-IR (KBr) *ν*_max_: 3275, 3107–2727, 1701, 1636–1449, 1229, 1084–980 cm^−1^; ^1^H-NMR: *δ* 12.47 (1H, bs, NOH), 11.52 (1H, bs, OH), 8.62 (1H, s, H-6), 7.72–7.70 (2H, d, *J* = 7.98 Hz, H-2', H-6'), 7.39–7.36 (2H, d, *J* = 8.16 Hz, H-3', H-5'), 6.00 (2H, s, CH_2_CO), 4.67 (2H, s, CH_2_OH), 2.51 (1H, s, CH_2_OH), 2.50 (3H, s, CH_3_). ^13^C-NMR: *δ* 190.70 (C, C=O), 152.50 (C, C-3), 137.21 (C, C-2), 135.11 (C, C-6), 135.10 (C, C-4), 130.44 (C, C-4'), 128.30 (C-2', C-6'), 128.26 (C, C-1'), 128.10, (C-3', C-5'), 127.82 (C, C-5), 59.67 (CH_2_CO), 58.44, (CH_2_OH), 13.25 (CH_3_); MS (*m/z*): 381.0 [M]^+^ (83), 299.3 (100), 281.4 (52.45), 269.2 (37.06), 161.0 (47.55), 106.0 (31.46); Anal. Calcd. for C_16_H_17_N_2_O_4_Br: C 50.41, H 4.49, N 7.35, Br 20.96; Found: C 50.58, H 4.33, N 7.48, Br 20.93%.

*3-Hydroxy-4-hydroxyiminomethyl-5-hydroxymethyl-2-methyl-1-(4-methylphenacyl)pyridinium bromide* (**5**). Yellow–light brown solid; R_f_ value: 0.22 (chloroform‒methanol, 6:1.5, v/v); mp after crystallization from methanol 240–241.5 °C; FT-IR (KBr) *ν*_max_: 3385, 3064–2853, 1684, 1636–1541, 1234, 1088–980 cm^−1^; ^1^H-NMR: *δ* 12.87 (1H, bs, NOH), 8.67 (1H, bs, OH), 8.62 (1H, s, H-6), 8.03–8.00 (2H, d, *J* = 8.22 Hz; H-3', H-5'), 7.48–7.46 (2H, d, *J* = 8.07 Hz, H-2', H-6'), 6.42 (2H, s, CH_2_CO), 4.81 (2H, s, CH_2_OH), 2.51, (1H, s, CH_2_OH), 2.49 (3H, s, CH_3_), 2,23 (3H, s, CH_3_). ^13^C-NMR: *δ* 190.06 (C, C=O), 152.49 (C, C-3), 145.50 (C, C-2), 145.31 (C, C-6), 137.24 (C, C-4), 135.19 (C, C-4'), 129.34 (C-2', C-6'), 128.59 (C, C-1'), 128.10 (C-3', C-5'), 127.80 (C, C-5), 64.42 (CH_2_CO), 58.43 (CH_2_OH), 13.25 (CH_3_); MS (*m/z*): 395.2 [M]^+^ (100), 313.0 (44.37), 295.5 (21.83), 264.8 (22.54), 162.1 (33.09), 132.9 (22.54); Anal. Calcd. for C_17_H_19_N_2_O_4_Br: C 51.66, H 4.85, N 7.09, Br 20.22; Found: C 51.65, H 4.83, N 7.27%.

*3-Hydroxy-4-hydroxyiminomethyl-5-hydroxymethyl-2-methyl-1-(4'-nitrophenacyl)pyridinium bromide* (**6**). Reddish brown solid; R_f_ value: 0.16 (chloroform‒methanol, 6:1.5, v/v); mp after crystallization from ethyl acetate 235–237 °C; FT-IR (KBr) *ν*_max_: 3347, 3069–2862, 1715, 1604–1528, 1217, 1032–1001 cm^−1^; ^1^H-NMR: *δ* 12.54 (1H, bs, NOH), 8.66 (1H, bs, OH), 8.62 (1H, s, H-6), 8.59–8.53 (2H, d, *J* = 8.10 Hz, H-3', H-5'), 8.47–8.39 (2H, d, *J* = 8.12 Hz, H-2', H-6'), 6.51 (2H, s, CH_2_CO), 4.82 (2H, s, CH_2_OH), 4.67 (1H, bs, CH_2_OH), 2.29 (3H, s, CH_3_); ^13^C-NMR: *δ* 190.12 (C, C=O), 152.55 (C, C-3), 150.68 (C, C-2), 146.90 (C, C-6), 145.60 (C, C-4), 145.51 (C, C-4'), 138.59 (C, C-1'), 138.23 (C, C-2', C-6'), 137.36 (C-3', C-5'), 130.05 (C, C-5), 64.68 (CH_2_CO), 58.54 (CH_2_OH), 13.34 (CH_3_); MS (*m/z*): 425.1 [M]^+^ (5.63), 344.0 (100), 326.1 (31.69), 314.3 (21.13), 164.0 (52.81), 161.0 (36.62), 106,7 (26.76); Anal. Calcd. for C_16_H_16_Br N_3_O_6_: C 45.09, H 3.78, N 9.86, Br 18.75; Found: C 44.70, H 4.00; N 9.67%.

*1-(4-Fluorophenacyl)-3-hydroxy-4-hydroxyiminomethyl-5-hydroxymethyl-2-methylpyridinium bromide* (**7**). Brown solid; R_f_ value: 0.31 (chloroform‒methanol, 6:1.5, v/v); mp after crystallization from ethyl acetate 225–225,5 °C; FT-IR (KBr) *ν*_max_: 3447, 3064–2359, 1699, 1599–1456, 1233, 1063–1001 cm^−1^; ^1^H-NMR: *δ* 12.95 (1H, bs, NOH), 8.66 (1H, bs, OH), 8.56 (1H, s, H-6), 8.21–8.18 (2H, d, *J* = 8.82 Hz, H-2', H-6'), 7.52-7.49 (2H, d, *J* = 8.82 Hz, H-3', H-5'), 6.26 (2H, s, CH_2_CO), 5.38 (2H, s, CH_2_OH), 4.67 (1H, s, CH_2_OH), 2.27 (3H, s, CH_3_). ^13^C-NMR *δ* 189.31 (C, C=O), 164.17 (C, C-3), 145.50 (C, C-2), 145.44 (C, C-6), 137.29 (C, C-4), 131.73 (C, C-4'), 131.62 (C-2', C-6'), 130.30 (C, C-1'), 128.13 (C-3', C-5'), 128.10 (C, C-5), 64.32 (CH_2_CO), 58.49 (CH_2_OH), 13.25 (CH_3_); MS (*m/z*): 399.2 [M]^+^ (53.52), 319.1 (9.86), 317.25 (100), 299.1 (30.28), 287.3 (36.62), 137.0 (32.39), 133.0 (36.62); Anal. Calcd. for C_16_H_16_BrFN_2_O_4_: C 48.14, H 4.04, N 7.02, Br 20.02; Found: C 47.53, H 4.22, N 6.95%.

*3-Hydroxy-4-hydroxyiminomethyl-5-hydroxymethyl-2-methyl-1-(4-methoxyphenacyl)pyridinium bromide* (**8**). Light brown solid; R_f_ value: 0.21 (chloroform‒methanol, 6:1.5, v/v); mp after crystallization from ethyl acetate 267–268 °C; FT-IR (KBr) *ν*_max_: 3383, 3042–2843, 1676, 1638–1516, 1244, 1045–980 cm^−1^; ^1^H-NMR: *δ* 13.00 (1H, bs, NOH), 8.66 (1H, bs, OH), 8.56 (1H, s, H-6), 8.13 (2H, s, CH_2_CO), 8.08- 8.05 (2H, d, *J* = 8.34 Hz; H-3', H-5'), 7.20 – 7.17 (2H, d, *J* = 8.97 Hz; H-2', H-6'), 6.55 (2H, s, CH_2_OH), 4.83 (1H, bs, CH_2_OH), 3.91 (3H, s, OCH_3_), 2.51 (3H, s, CH_3_). ^13^C-NMR: *δ* 188.73 (C, C=O), 164.42 (C, C-3), 145.52 (C, C-2), 145.33 (C, C-6), 137.25 (C, C-4), 135.11 (C, C-4'), 130,97 (C-2', C-6'), 128.05 (C, C-1'), 128.32 (C-3', C-5'), 127.80 (C, C-5), 64.10 (CH_2_CO), 58.47 (CH_2_OH), 13.21 (CH_3_); MS (*m/z*): 411.2 [M]^+^ (96.5), 331.0 (3.49), 329.35 (100), 311.0 (17.48), 299.1 (42.65), 134.0 (57.34), 106.0 (60.84); Anal. Calcd. for C_17_H_19_N_2_O_5_Br: C 49.65, H 4.66, N 6.81, Br 19.43; Found: C 49.79, H 4.46, N 6.83, Br 19.35%.

*3-Hydroxy-4-hydroxyiminomethyl-5-hydroxymethyl-2-methyl-1-(4'-phenylphenacyl)pyridinium bromide* (**9**). Yellow solid; R_f_ value: 0.39 (chloroform‒methanol, 6:1.5, v/v); mp after crystallization from methanol 227–228 °C; FT-IR (KBr) *ν*_max_: 3397, 3075–2789, 1691, 1604–1451, 1237, 1088–995 cm^−1^; ^1^H-NMR: *δ* 13.02 (1H, bs, NOH), 12.78 (1H, bs, OH), 8.64 (1H, s, H-6), 8.19–8.166 (2H, d, *J* = 8.43 Hz, H-3', H-5'), 8.00–7.97 (2H, d, *J* = 8.43 Hz, H-2', H-6'); 7.58–7.45 (5H, m, Ph), 6.64 (2H, s, CH_2_CO), 4.81 (2H, s, CH_2_OH); 4.75 (1H, bs, CH_2_OH), 2.50 (3H, s, CH_3_); ^13^C-NMR: *δ* 190.13 (C, C=O), 152.46 (C, C-3), 146.02 (C, C-2), 145.49 (C, C-4), 145.38 (C, C-6), 142.86 (C, C-4'), 138.45 (C-2', C-6'), 138.05 (C, C-1'), 137.30 (C-3', C-5'), 136.83 (C, C-5), 64.40 (CH_2_CO), 58.48 (CH_2_OH), 13,22 (CH_3_); MS (*m/z*): 457.1 [M]^+^ (100), 375.3 (87.41), 357.3 (15.38), 345.0 (31.12), 164.3 (62,23); Anal. Calcd. for C_22_H_21_N_2_O_4_Br: C 57.78, H 4.63, N 6.13, Br 17.47; Found: C 57.63, H 4.46, N 6.03%.

*3-Hydroxy-4-hydroxyiminomethyl-5-hydroxymethyl-2-methyl-1-(2-methoxyphenacyl)pyridinium bromide* (**10**). Brown solid; R_f_ value: 0.36 (chloroform‒methanol, 6:1.5, v/v); mp after crystallization from methanol 217–219 °C; FT-IR (KBr) *ν*_max_: 3309, 3102–2949, 1674, 1597–1437, 1252, 1055–980 cm^−1^; ^1^H-NMR: *δ* 12.95 (1H, bs, NOH), 8.67 (1H, bs, OH), 8.61 (1H, s, H-6), 7.86–7.84 (2H, d, *J* = 6.03 Hz H-3', H-5'), 7.36–7.33 (2H, d, *J* = 8.40 Hz H-2', H-6'), 7.66 (2H, s, CH_2_CO), 6.53 (1H, bs, CH_2_OH), 4.80 (3H, s, OCH_3_), 4.04 (2H, s, CH_2_OH), 2,51 (3H, s, CH_3_); ^13^C-NMR: *δ* 190.26 (C, C=O), 159.98 (C, C-3), 145.58 (C, C-2), 145.51 (C, C-6), 145.19 (C, C-4), 137.09 (C, C-4'), 136.97 (C, C-1'), 136.27 (C-2', C-6'), 130.25 (C-3', C-5'), 128.00 (C, C-5), 67.86 (CH_2_CO), 58.45 (CH_2_OH), 13.19 (CH_3_); anal. C 49.65, H 4.66, N 6.81, Br 19.43% calcd for C_17_H_19_N_2_O_5_Br C 49.60, H 4.65, N 6.58%.

### 3.5. Crystal Structure Determination

Single crystal of **7** suitable for X-ray single crystal analysis was obtained at room temperature by partial evaporation from ethyl-acetate solution. The intensities were collected on a Oxford Diffraction Xcalibur2 diffractometer (Zagreb, Croatia) with a Sapphire 3 CCD detector using graphite-monochromated MoKα radiation (λ = 0.71073 Å) at 150 K. The *CrysAlisPro* [18] program was used for the data collection and processing. The intensities were corrected for absorption using the multi-scan absorption correction method [18]. The structure was solved by direct methods with *SIR2004* [19] and refined by full-matrix least-squares calculations based on F^2^ using *SHELXL-97* [20] integrated in *WinGX* [21] program package. Hydrogen atoms of oxygen O1, O2 and O3 atoms have been found in Fourier map and their coordinates and thermal isotropic parameters have been refined freely. All other hydrogen atoms were treated using appropriate riding models, with *SHELXL-97* defaults [20]. *PLATON* [22] and *Mercury* [23] programs were used for structure analysis and molecular and crystal structure drawings preparation. The CCDC 990857 contains the supplementary crystallographic data for this paper. These data can be obtained free of charge from The Cambridge Crystallographic Data Centre via www.ccdc.cam.ac.uk/data_request/cif. Crystal and refinement data for **7**: C_16_H_16_BrFN_2_O_4_, *M*r = 399.22; triclinic space group *P* 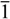 (No. 2); *a* = 6.7128(3), *b* = 8.1316(4), *c* = 15.9969(8) Å; *α* = 94.964(4), *β* = 92.224(4), *γ* = 111.877(5)°; *V* = 804.89(7) Å^3^; *Z* = 2; *d*_x_ = 1.647 g cm^−3^; *θ*_max_ = 27.0°; *R*_Int_ = 0.0290; *S* = 1.004; *R*[*I* ≥ 2*σ*(*I*)] = 0.0357, *wR*[all data] = 0.0991; 0.698 < ∆*ρ* < −0.760 eA^−3^.

## 4. Conclusions

In conclusion, a comparative study of the quaternization reaction of pyridoxal oxime with a series of substituted phenacyl bromides under microwave irradiation is reported in this paper. The preparation of quaternary pyridininium salts under microwave irradiation proved to be a fast, environmentally friendly, and facile method. The microwave irradiation provided a remarkable rate of acceleration for *N*-alkylation, reaction times decreased dramatically, the consumed energy decreased considerably, the yields are higher and this method could be considered as eco-friendly.

The MW method performed in acetone offered easier work up, cleaner products and higher yields. The solvent-free method was more eco-friendly because there was no solvent used in the synthesis, but the disadvantage is longer work up, use of solvents in the post-synthetic stage, and less cleaner products. One of the primary goals of the future experiment will be elimination of solvents from the post-synthetic stage after solventless quaternization. The X-ray structure analysis of **7** shows that the bond lengths and angles present no unexpected features, and that phenyl ring is rotated with respect to the pyridinium ring. Several types of intermolecular hydrogen bonds and one π...π interaction link cation and bromides into three-dimensional network. The continuation of this work to determine antibacterial, antifungal and antidotal activity of the novel synthesized compounds is in progress.

## Supplementary Materials

Supplementary materials can be accessed at: http://www.mdpi.com/1420-3049/19/6/7610/s1.
