# Supplementary material for: An Efficient Synthesis of Pyridoxal Oxime Derivatives under Microwave Irradiation
Source: Molecules. 2014 Jun 6;19(6):7610–20. doi: 10.3390/molecules19067610 (PMC6271574; doi:10.3390/molecules19067610)

Supplementary Materials

Figure S1. FT-IR spectrum of compound **2**.

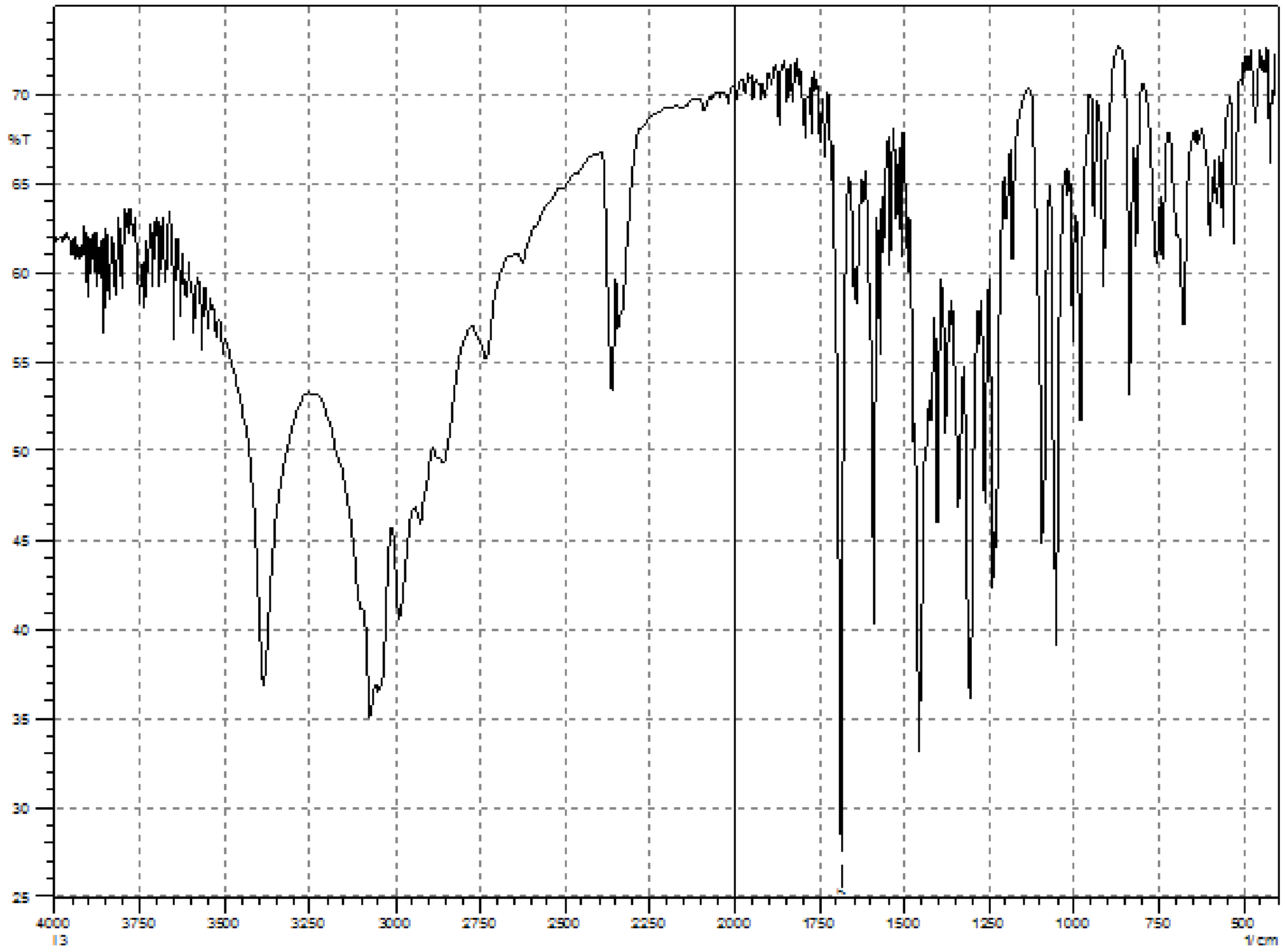

Figure S2. ^{1}H-NMR spectrum of compound **2** in DMSO-d_6 solution.

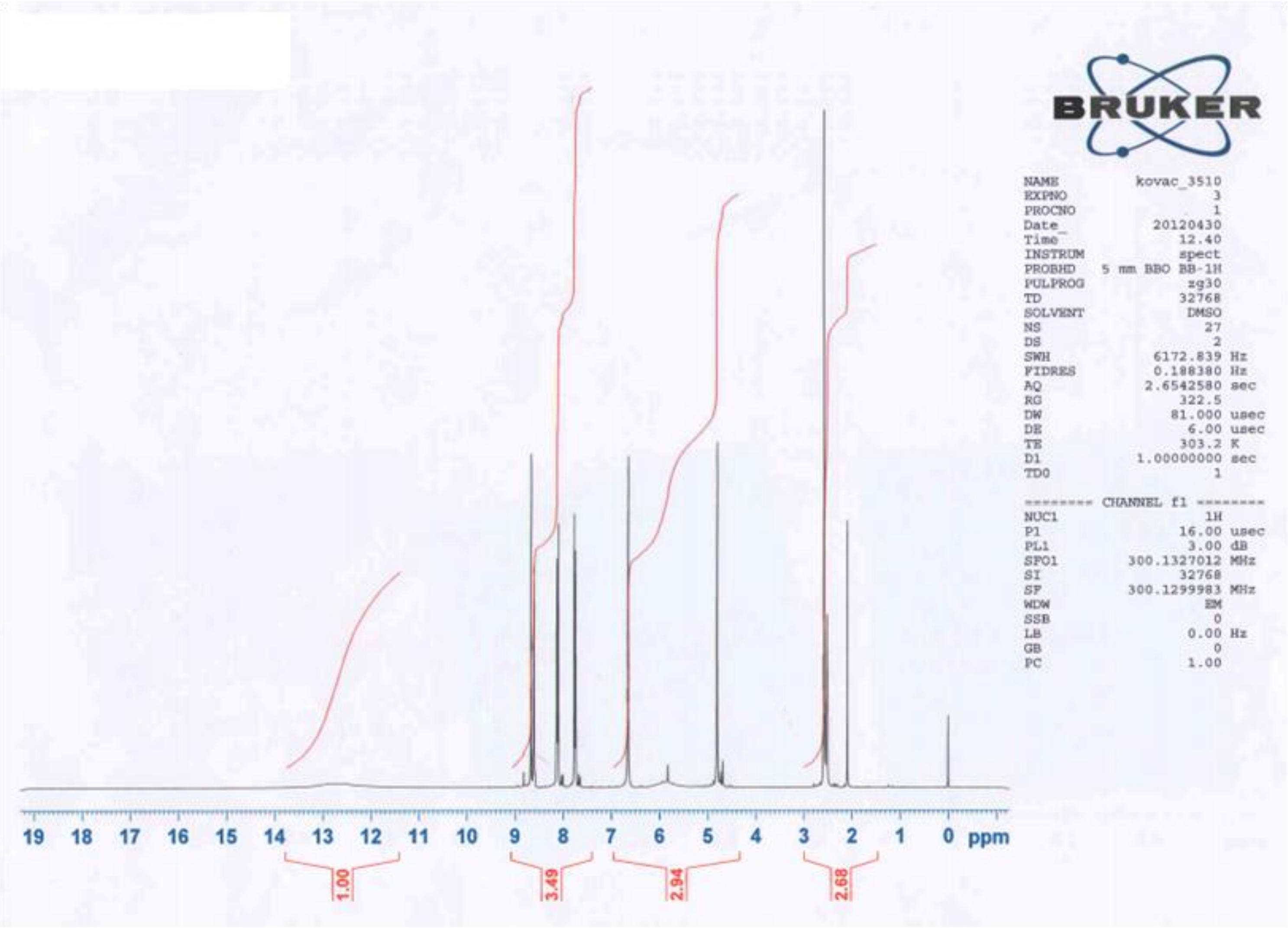

Figure S3. ^{13}C-NMR spectrum of compound **2** in DMSO-d_6 solution.

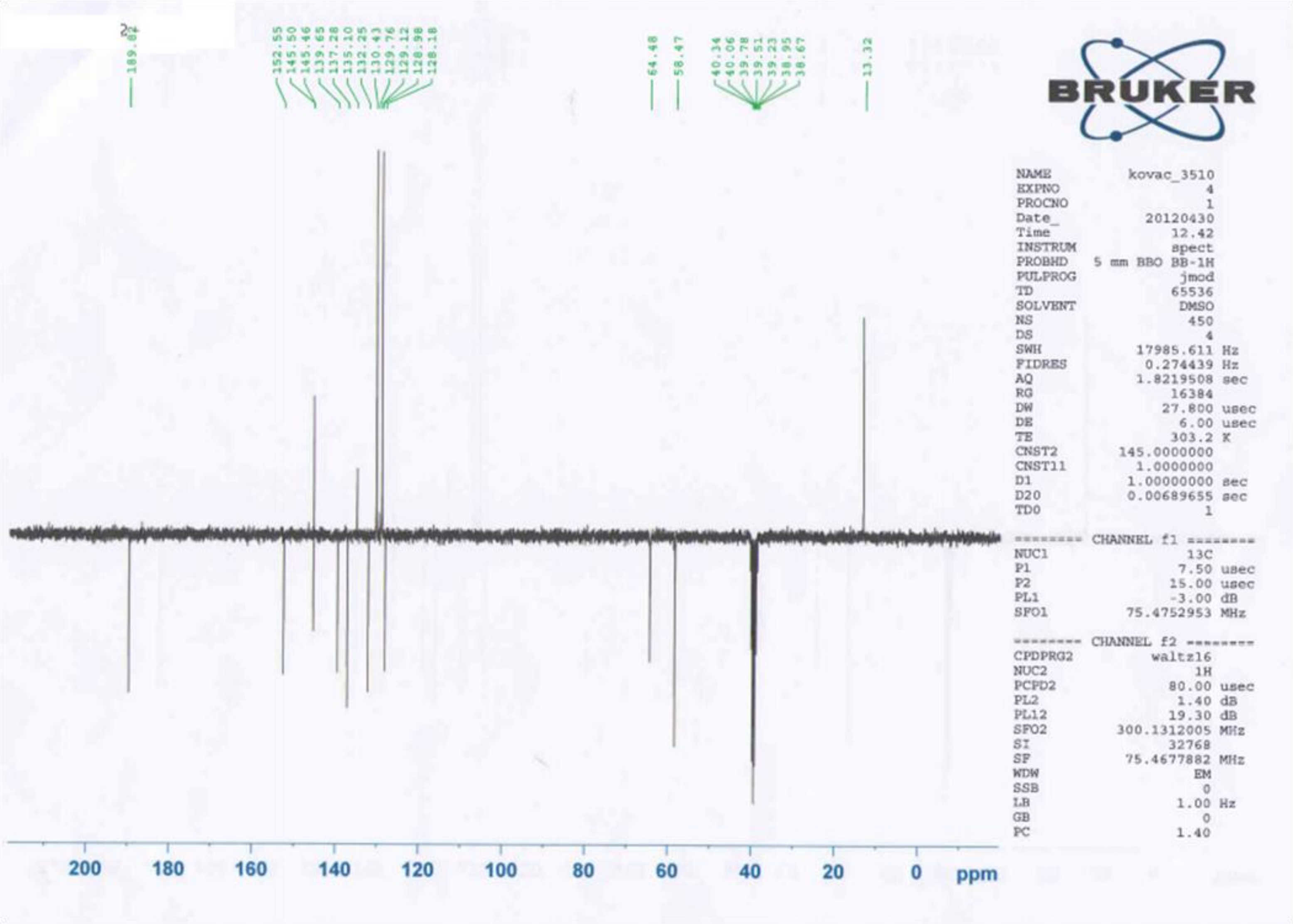

Figure S4. FT-IR spectrum of compound **3.**

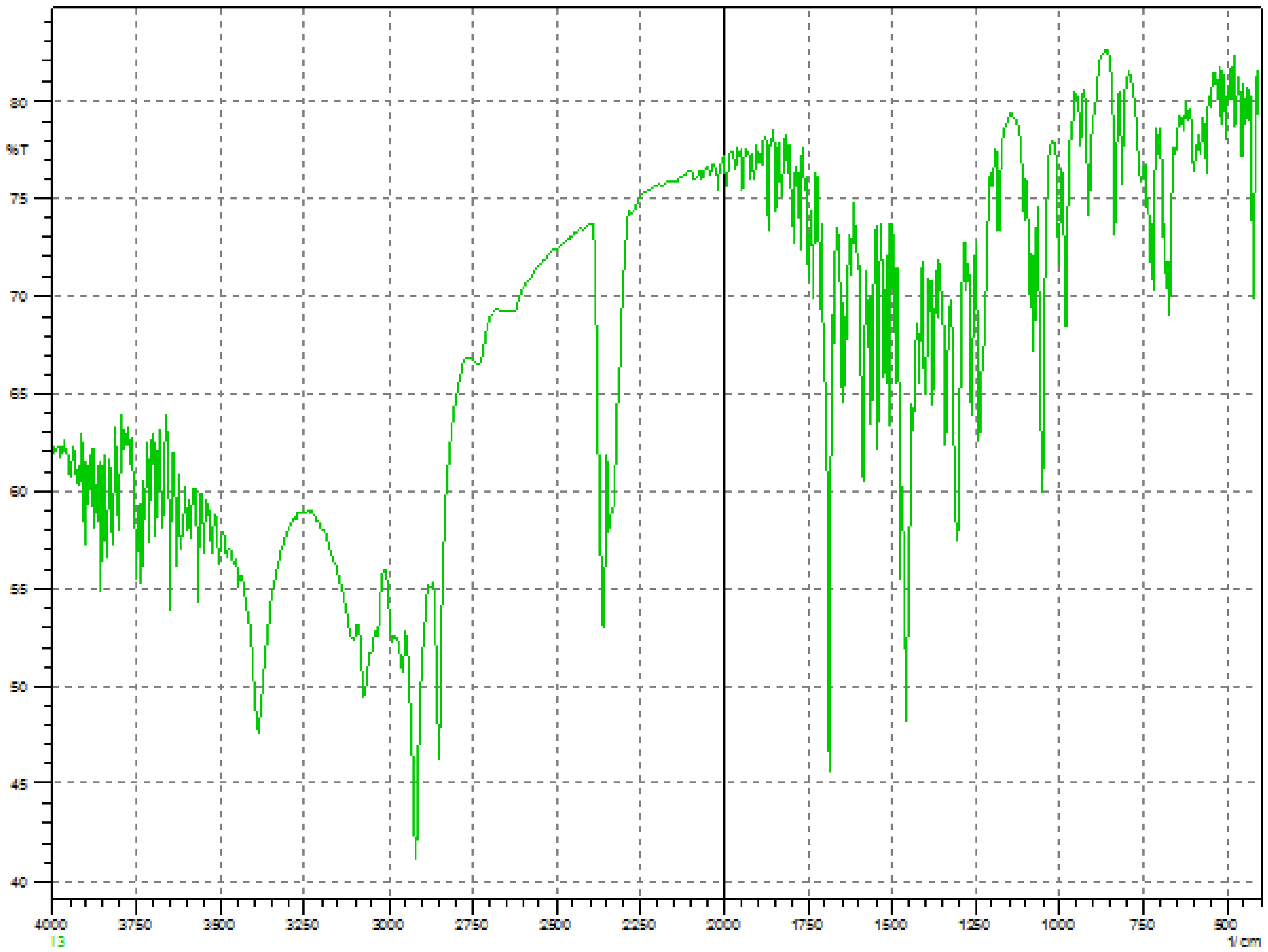

Figure S5. ^{1}H-NMR spectrum of compound **3** in DMSO-d_6 solution.

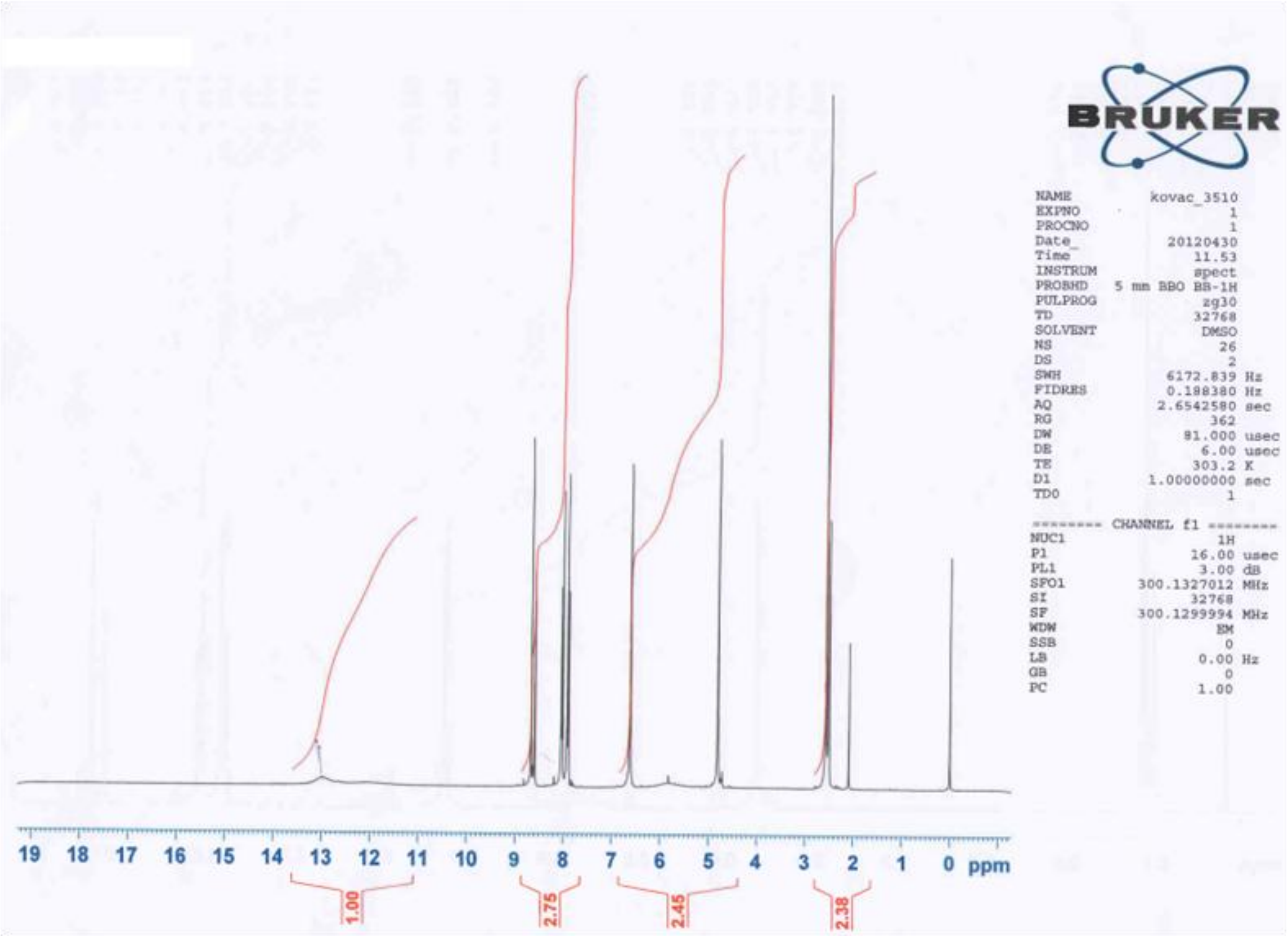

Figure S6. ^{13}C-NMR spectrum of compound **3** in DMSO-d_6 solution.

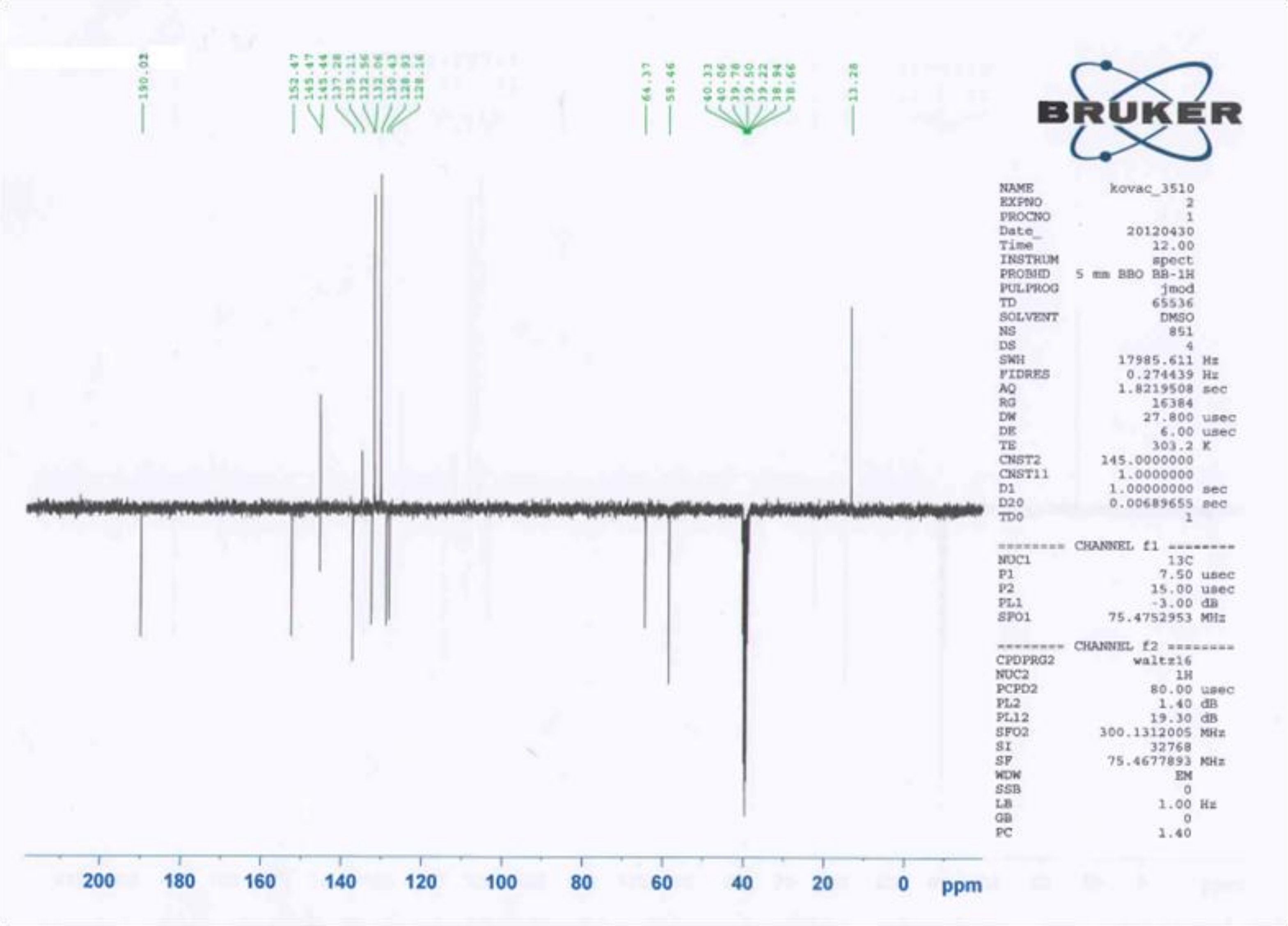

Figure S7. FT-IR spectrum of compound **4.**

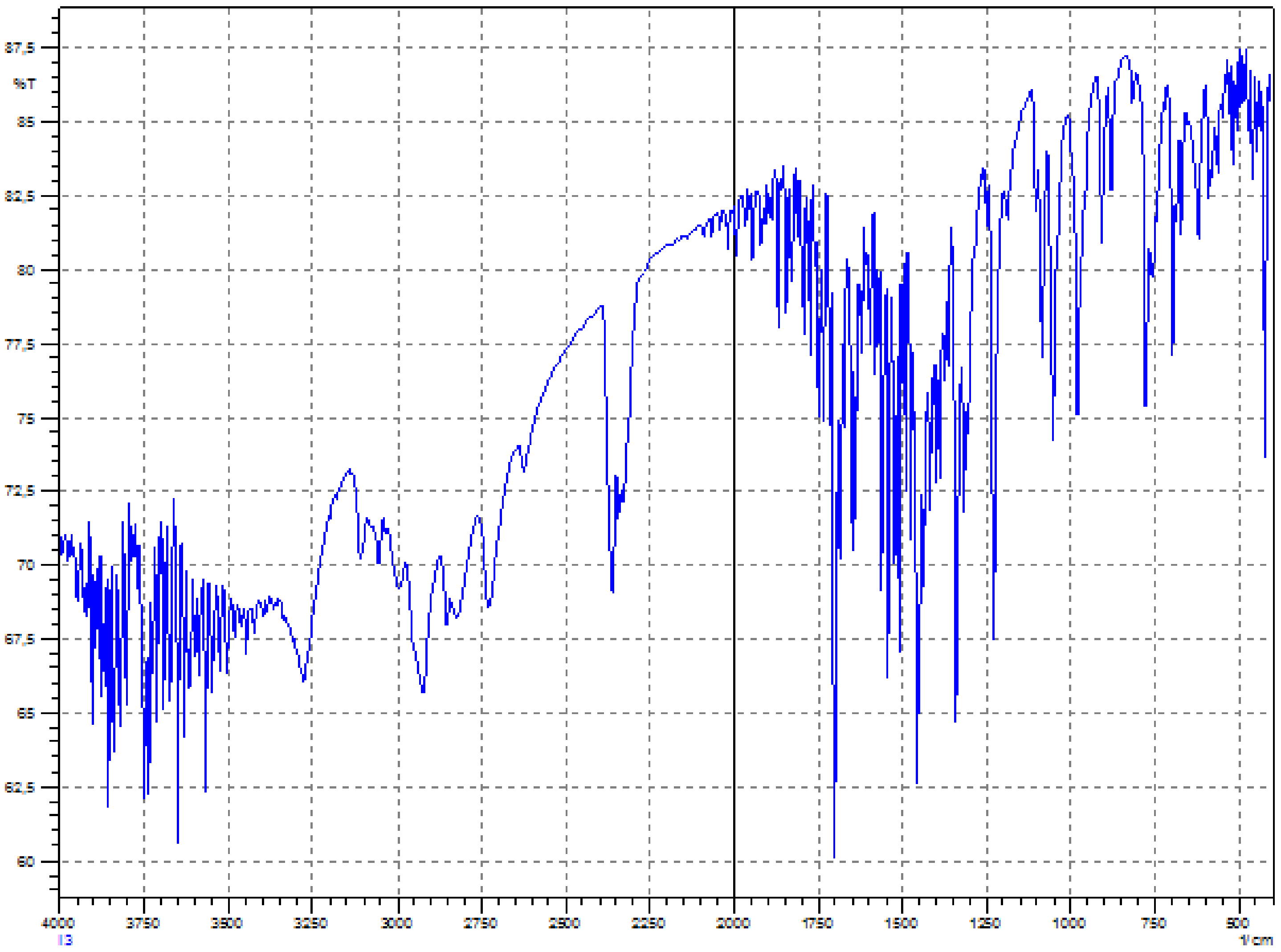

Figure S8. ^{1}H-NMR spectrum of compound **4** in DMSO-d_6 solution.

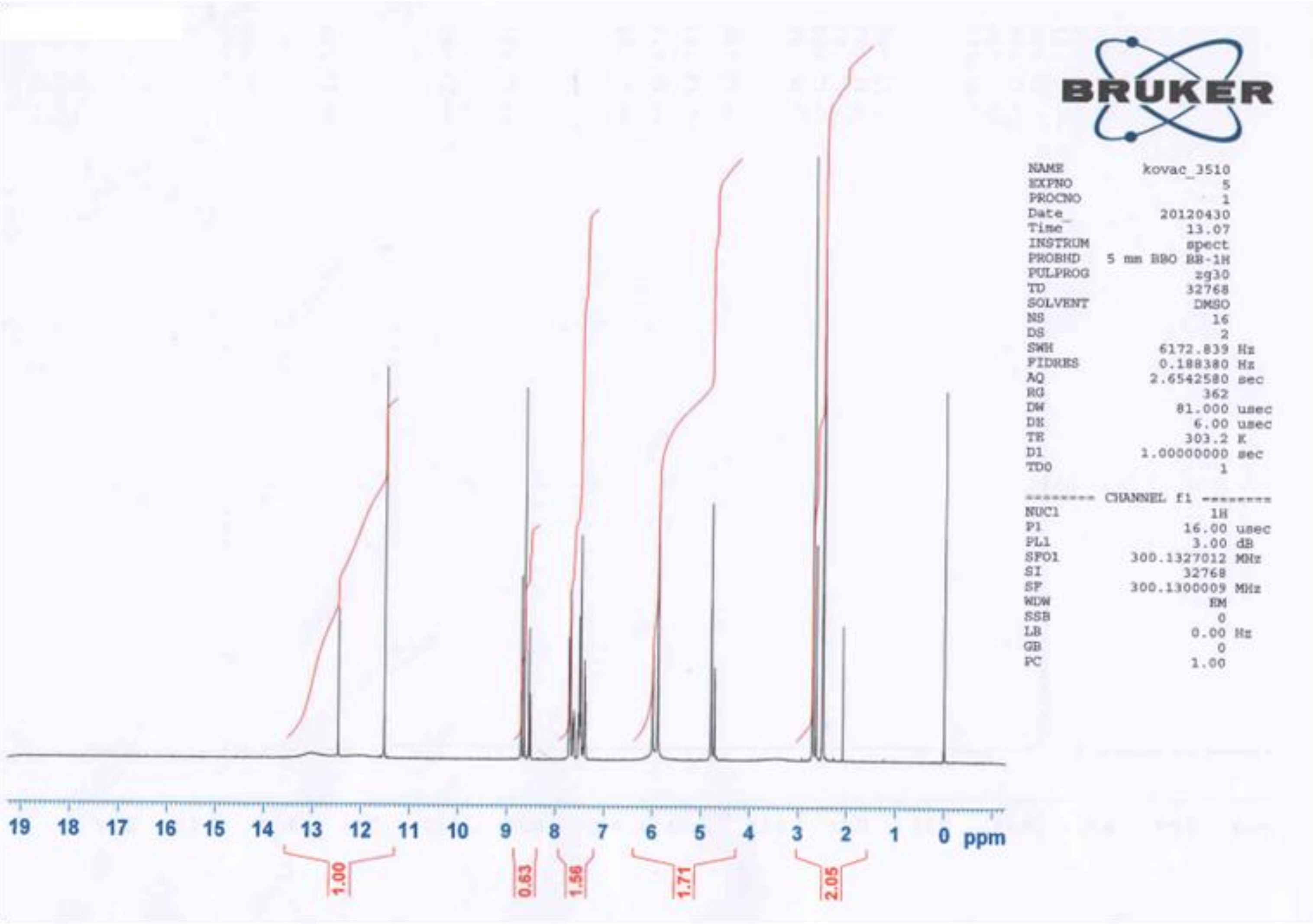

Figure S9. ^{13}C-NMR spectrum of compound **4** in DMSO-d_6 solution.

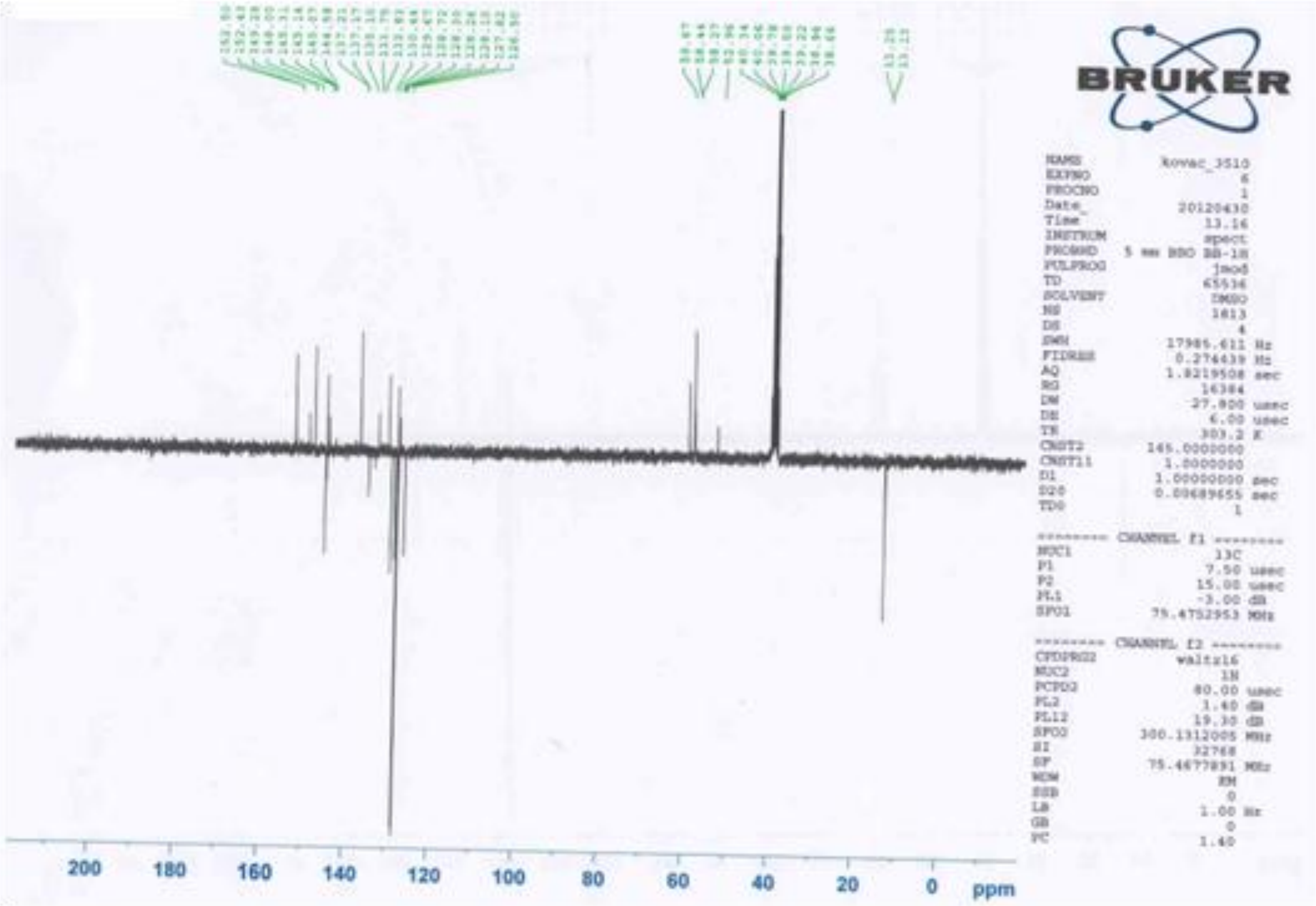

Figure S10. FT-IR spectrum of compound **5.**

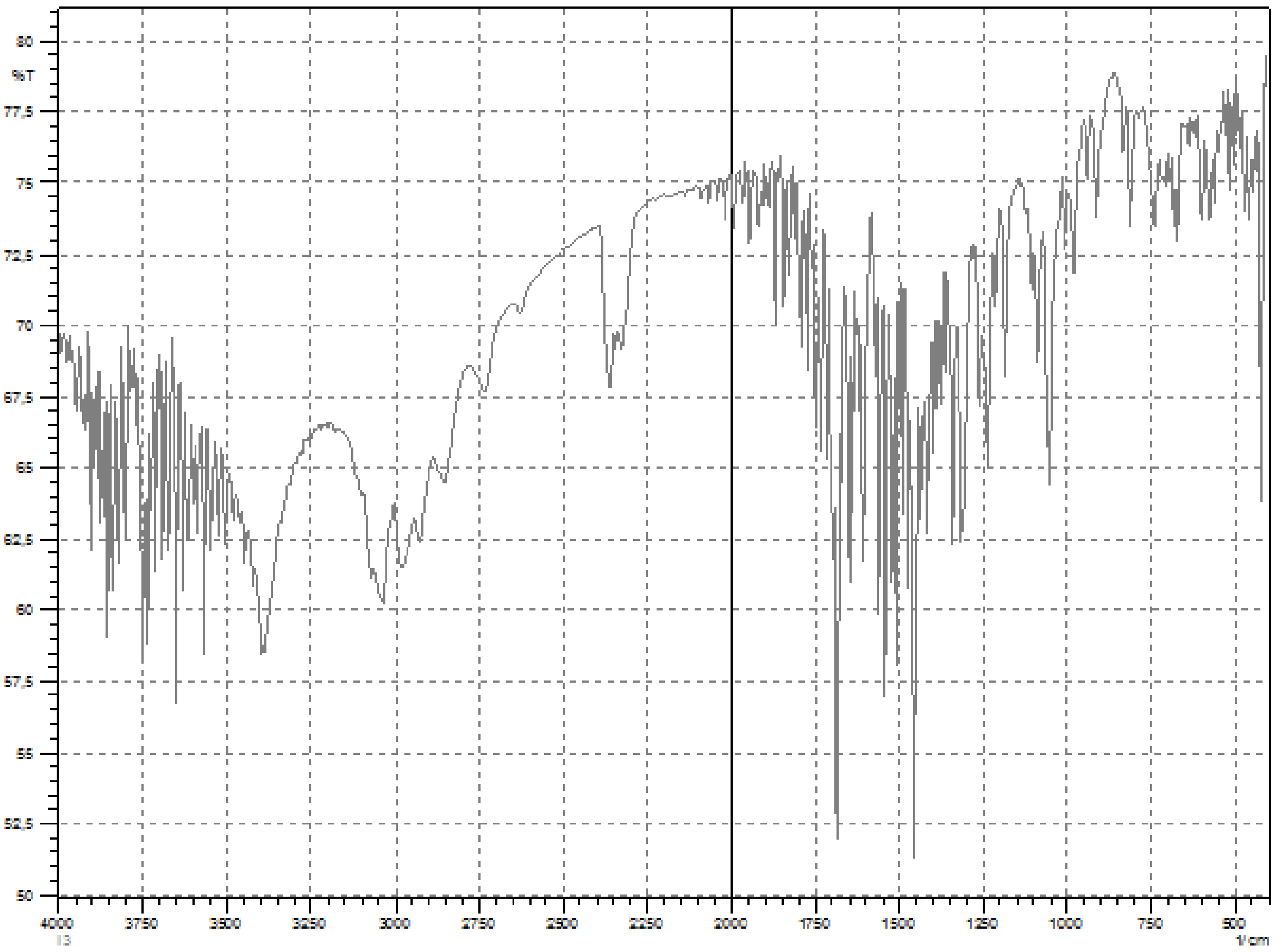

Figure S11. ^{1}H-NMR spectrum of compound **5** in DMSO-d_6 solution.

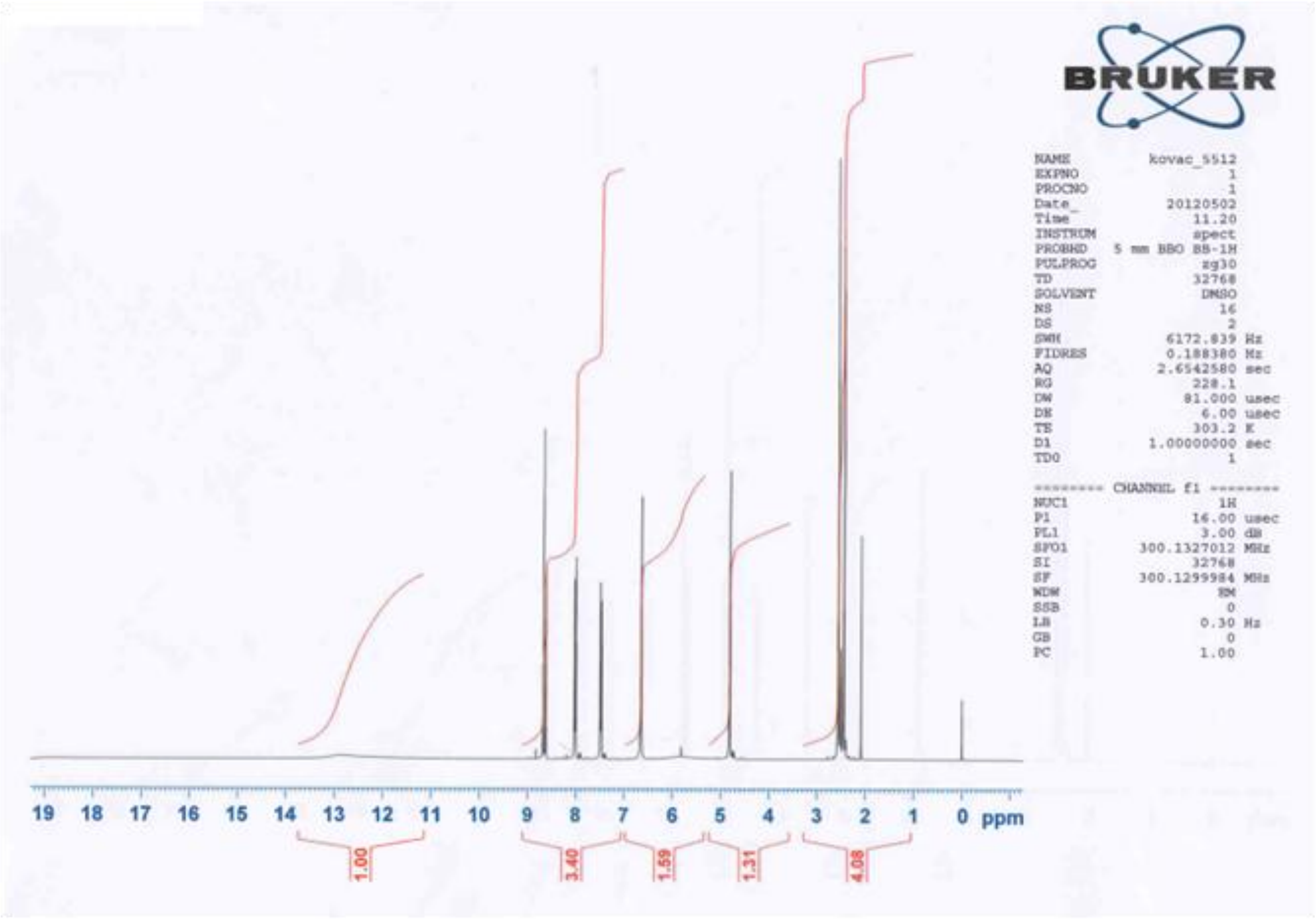

Figure S12. ^{13}C-NMR spectrum of compound **5** in DMSO-d_6 solution.

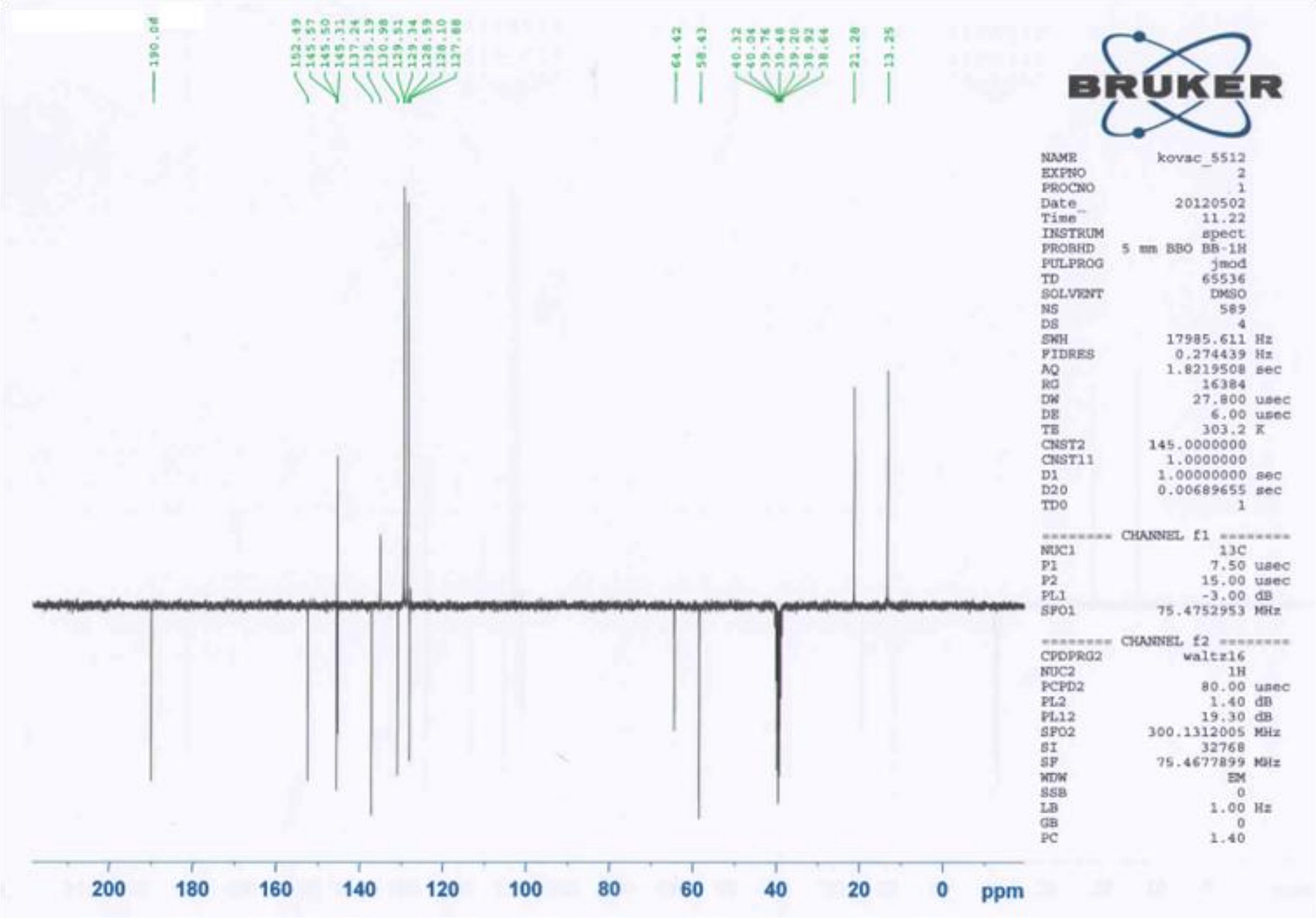

Figure S13. FT-IR spectrum of compound **6.**

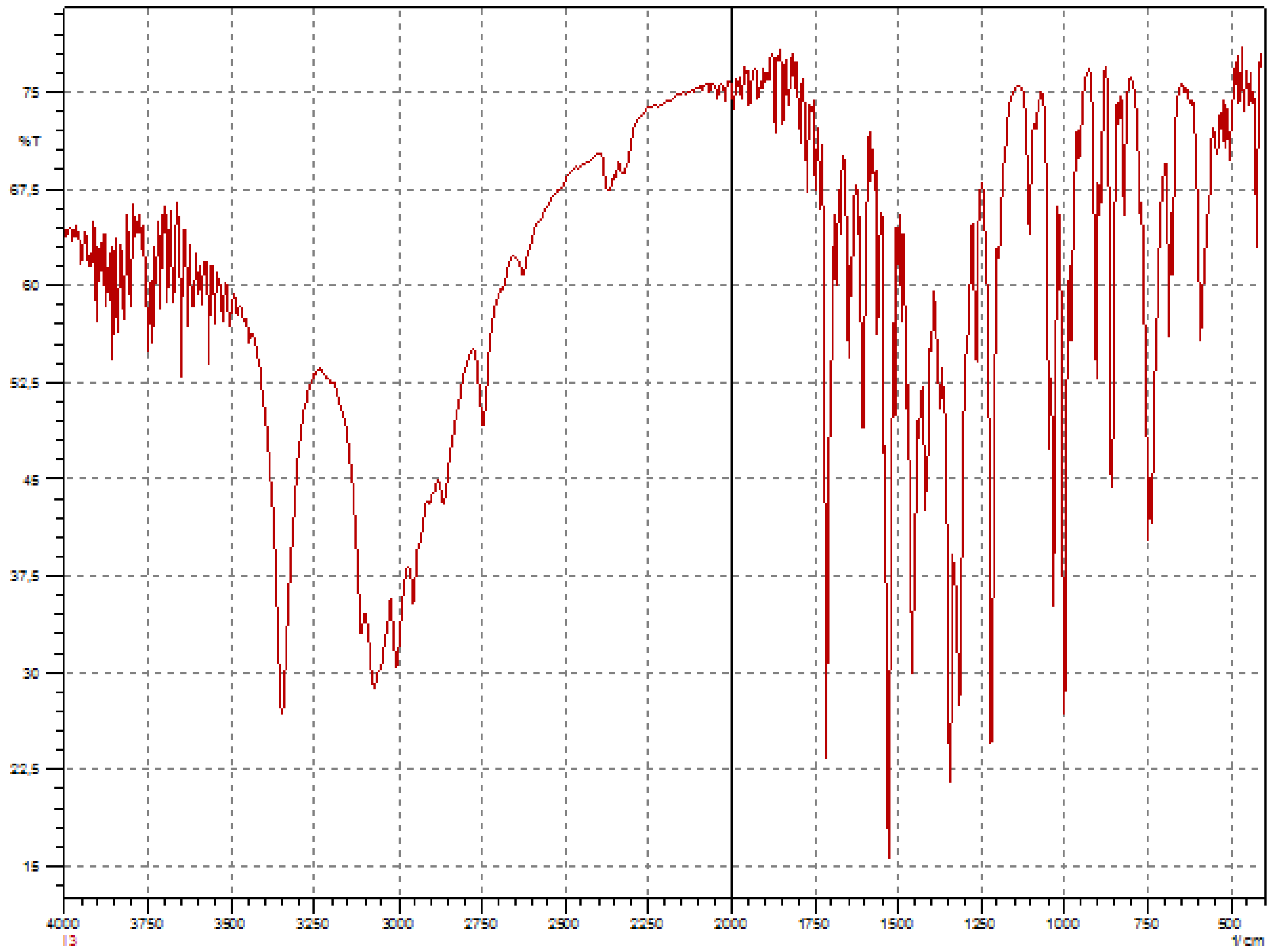

Figure S14. ^{1}H-NMR spectrum of compound **6** in DMSO-d_6 solution.

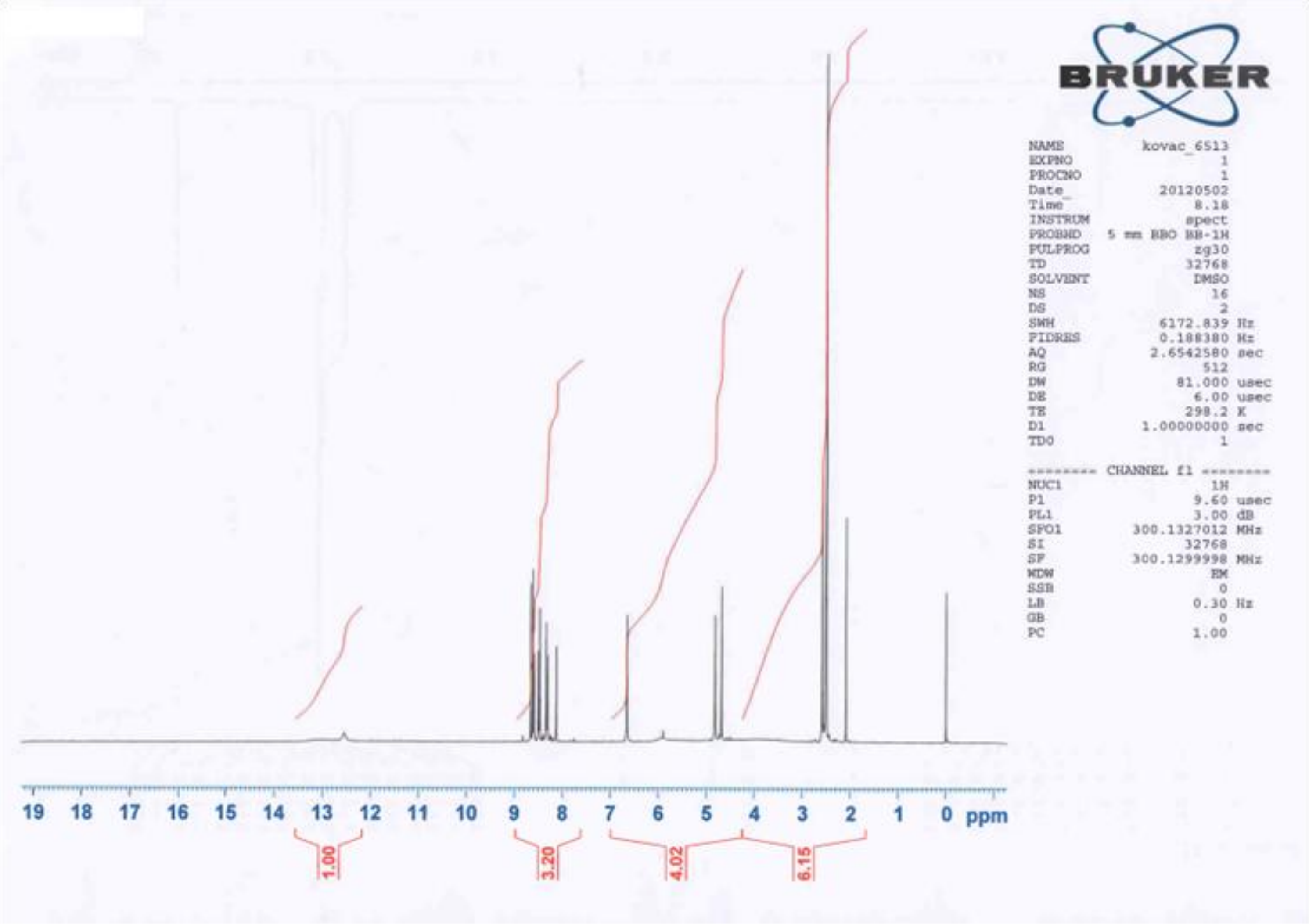

Figure S15. ^{13}C-NMR spectrum of compound **6** in DMSO-d_6 solution.

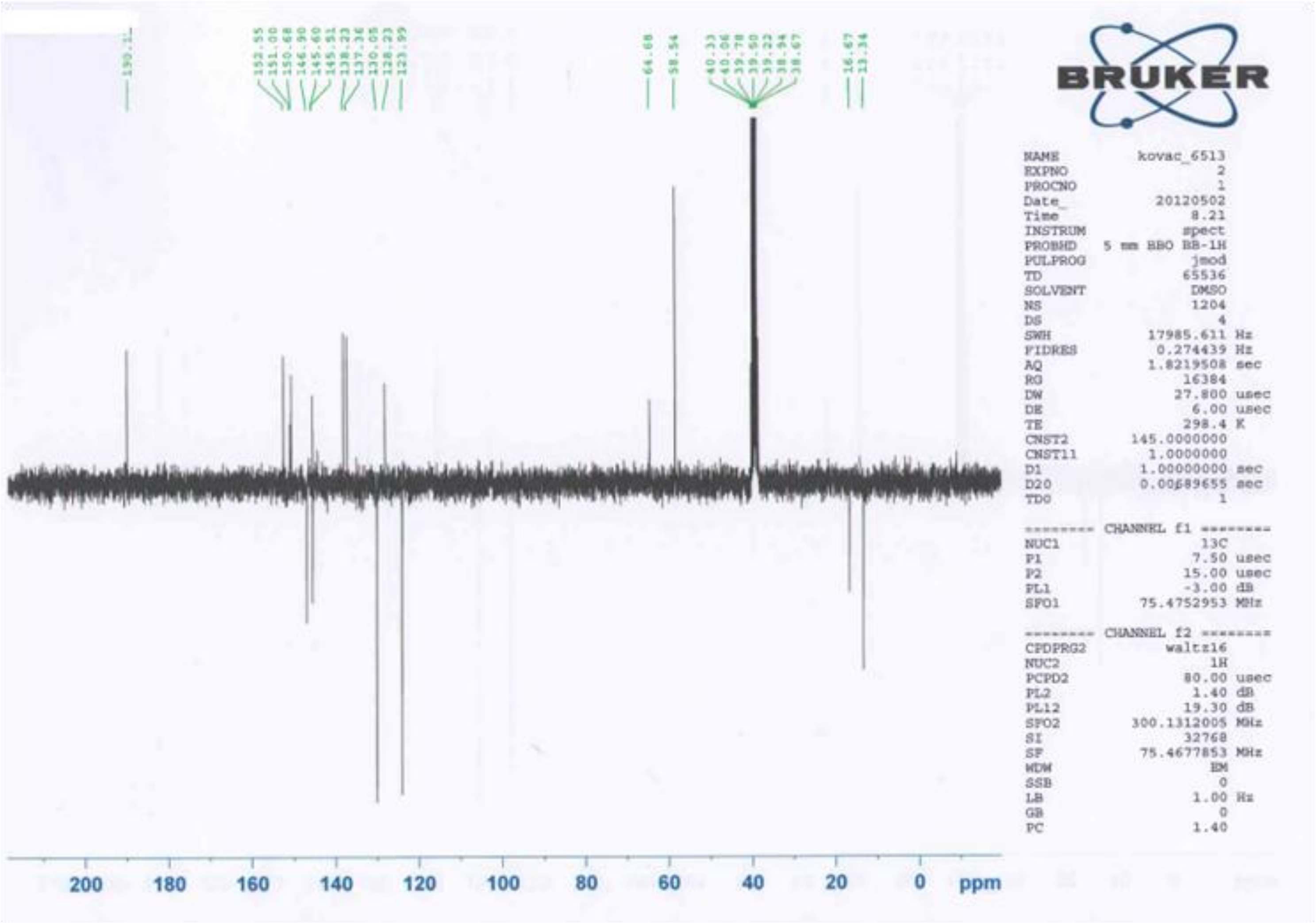

Figure S16. FT-IR spectrum of compound **7.**

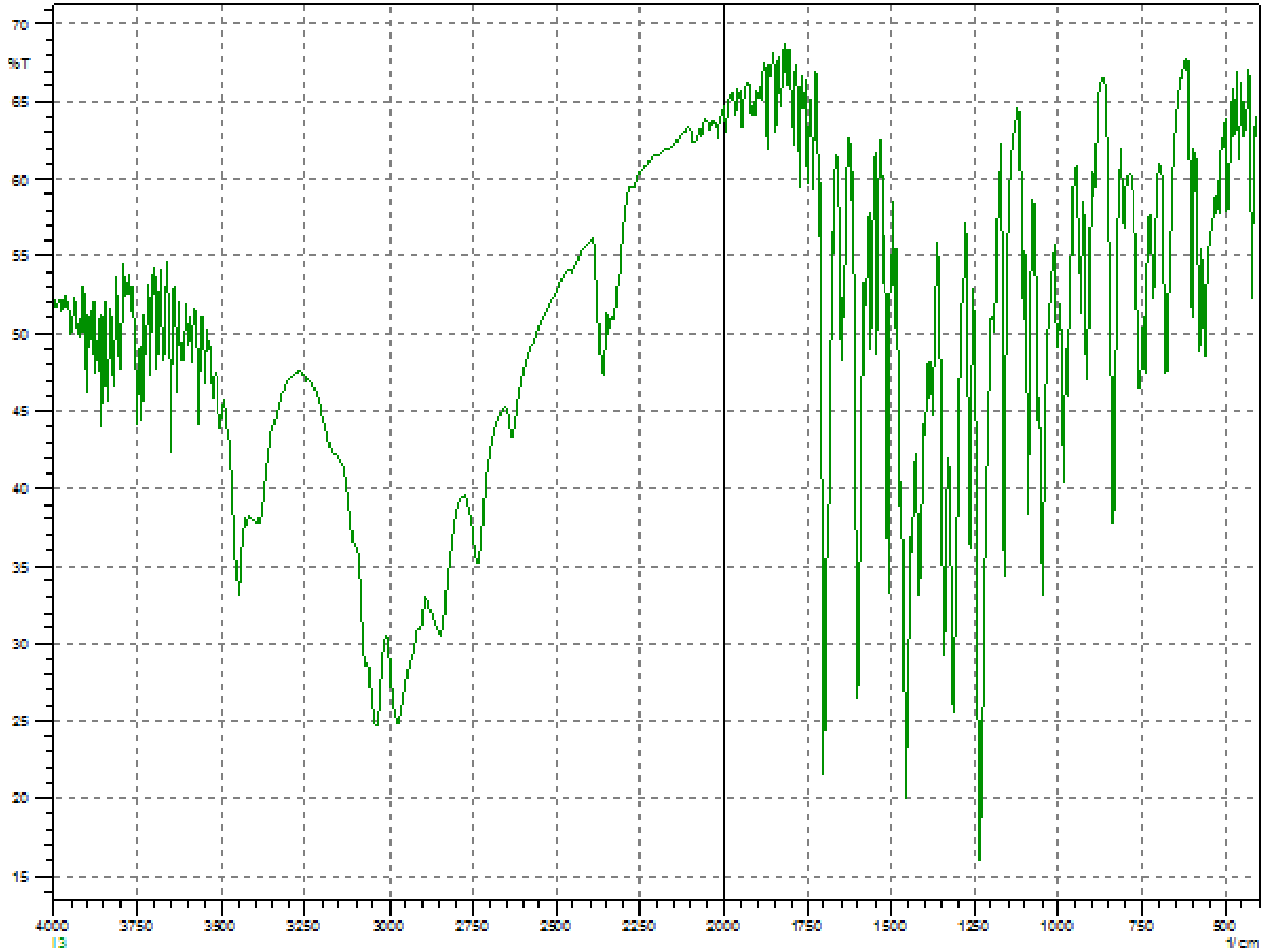

Figure S17. ^{1}H-NMR spectrum of compound **7** in DMSO-d_6 solution.

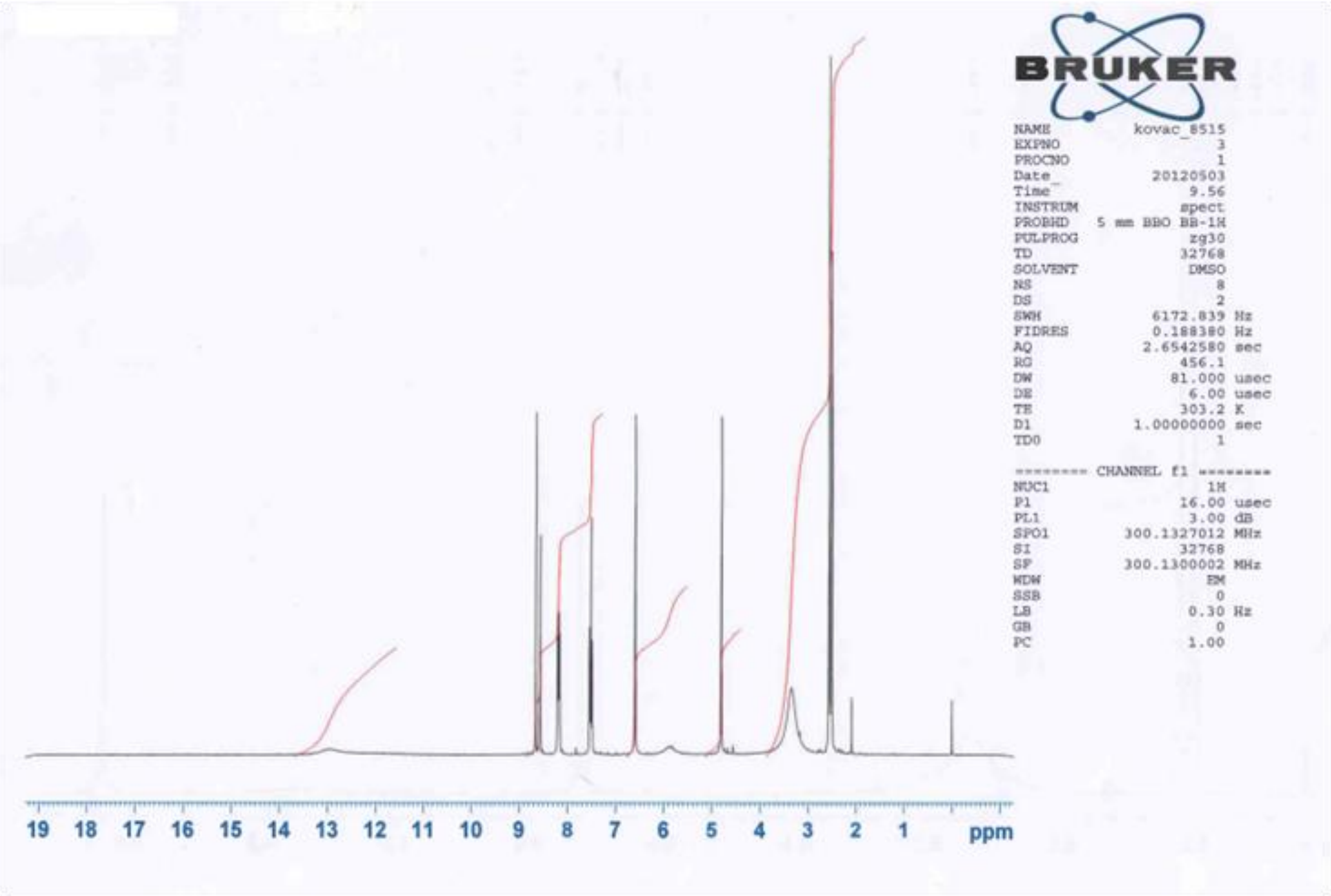

Figure S18. ^{13}C-NMR spectrum of compound **7** in DMSO-d_6 solution.

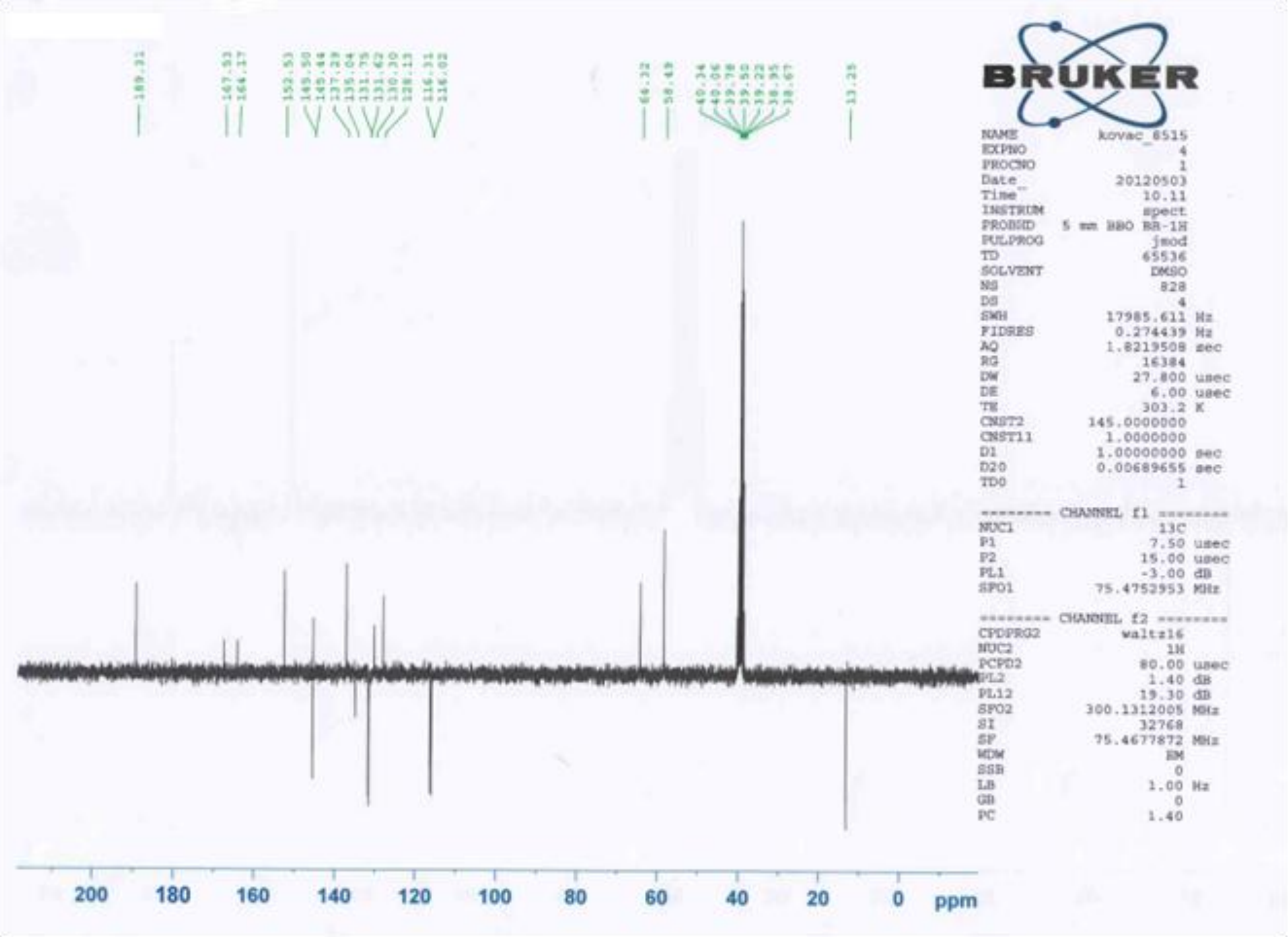

Figure S19. FT-IR spectrum of compound **8.**

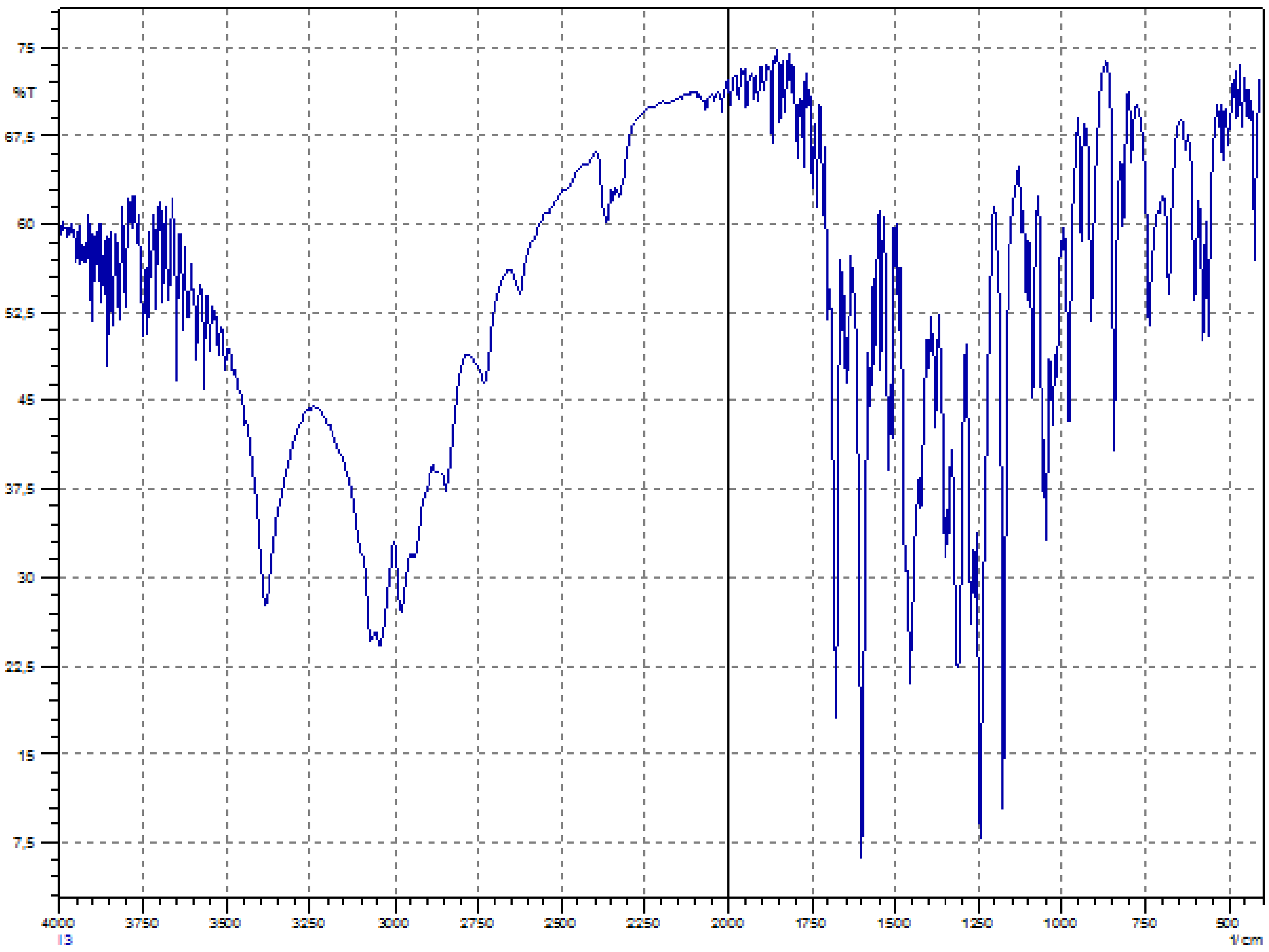

Figure S20. ^{1}H-NMR spectrum of compound **8** in DMSO-d_6 solution.

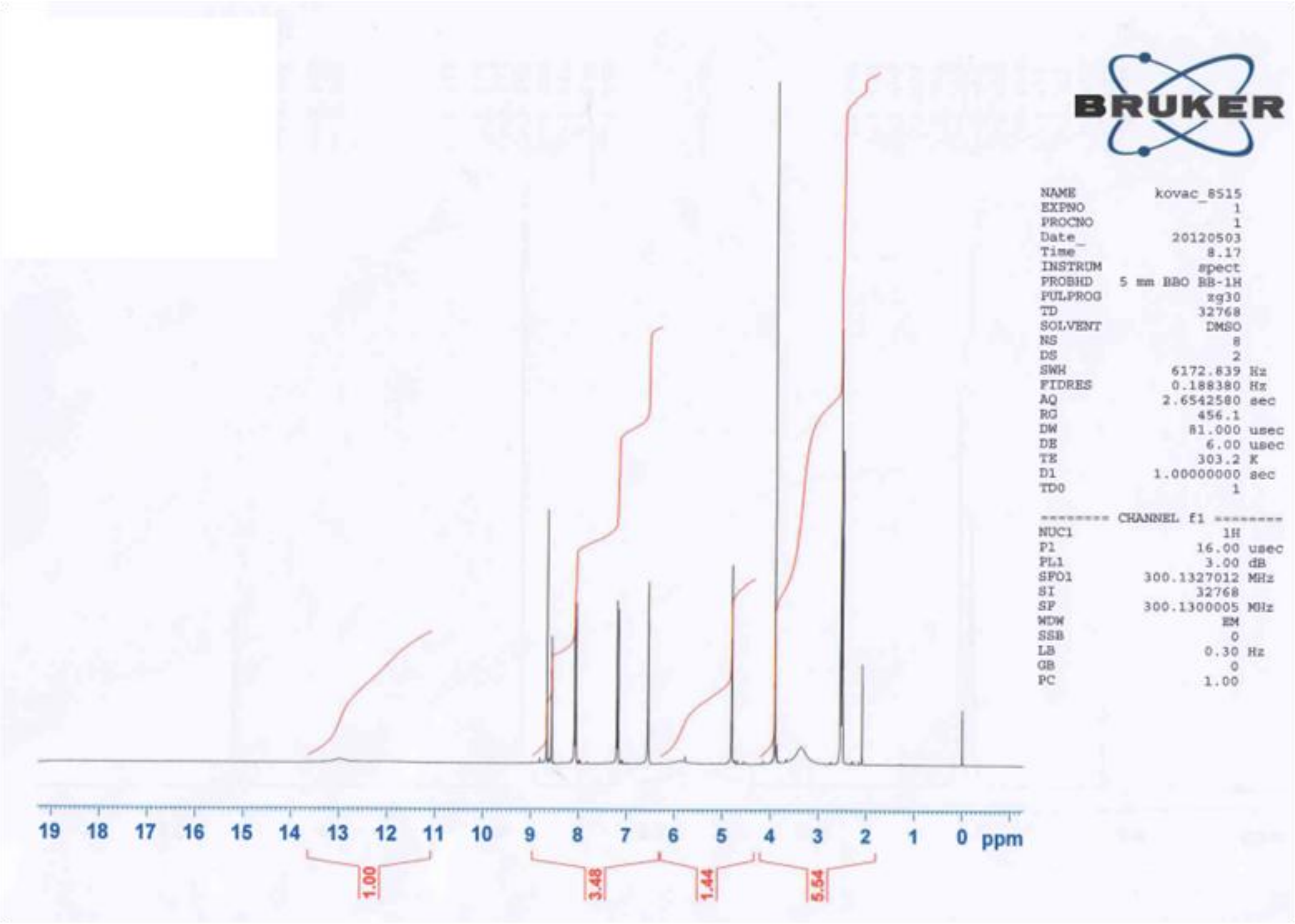

Figure S21. ^{13}C-NMR spectrum of compound **8** in DMSO-d_6 solution.

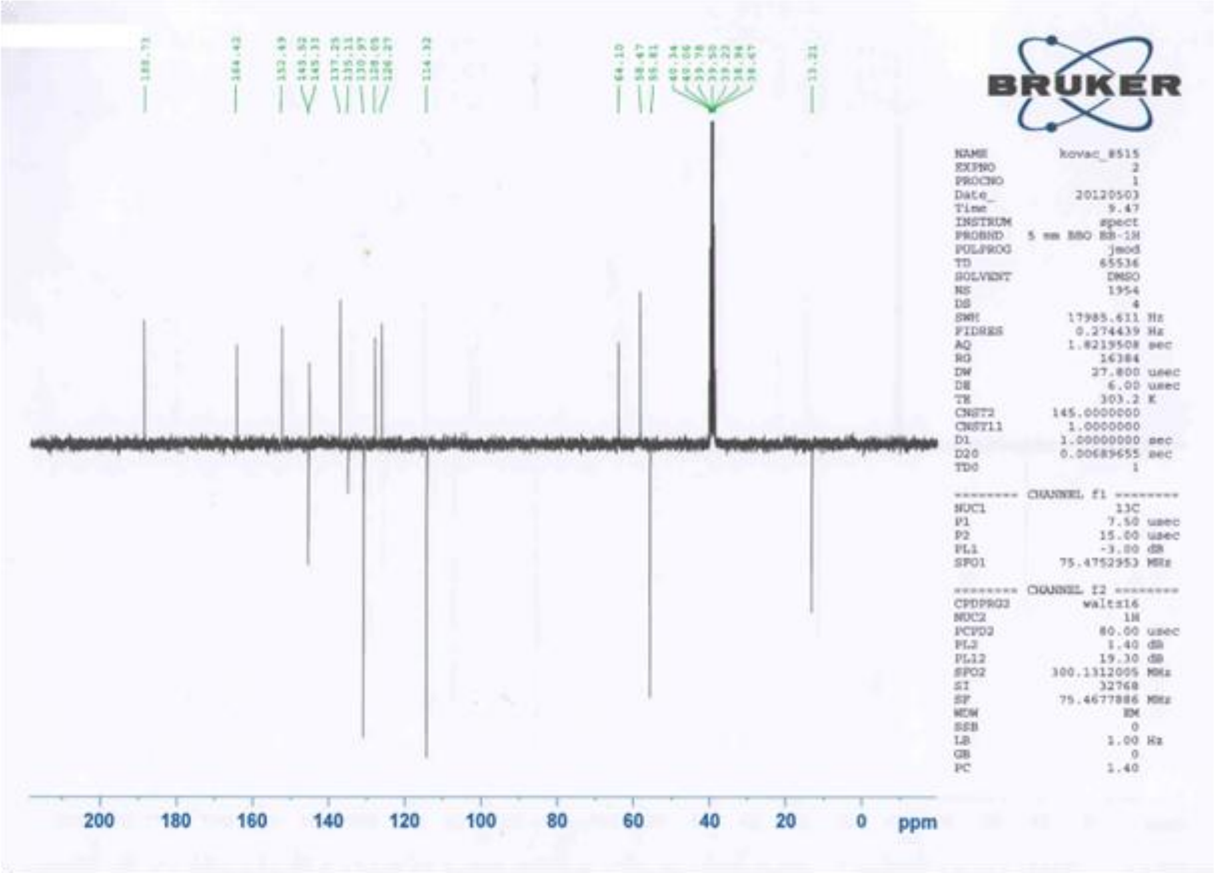

Figure S22. FT-IR spectrum of compound **9.**

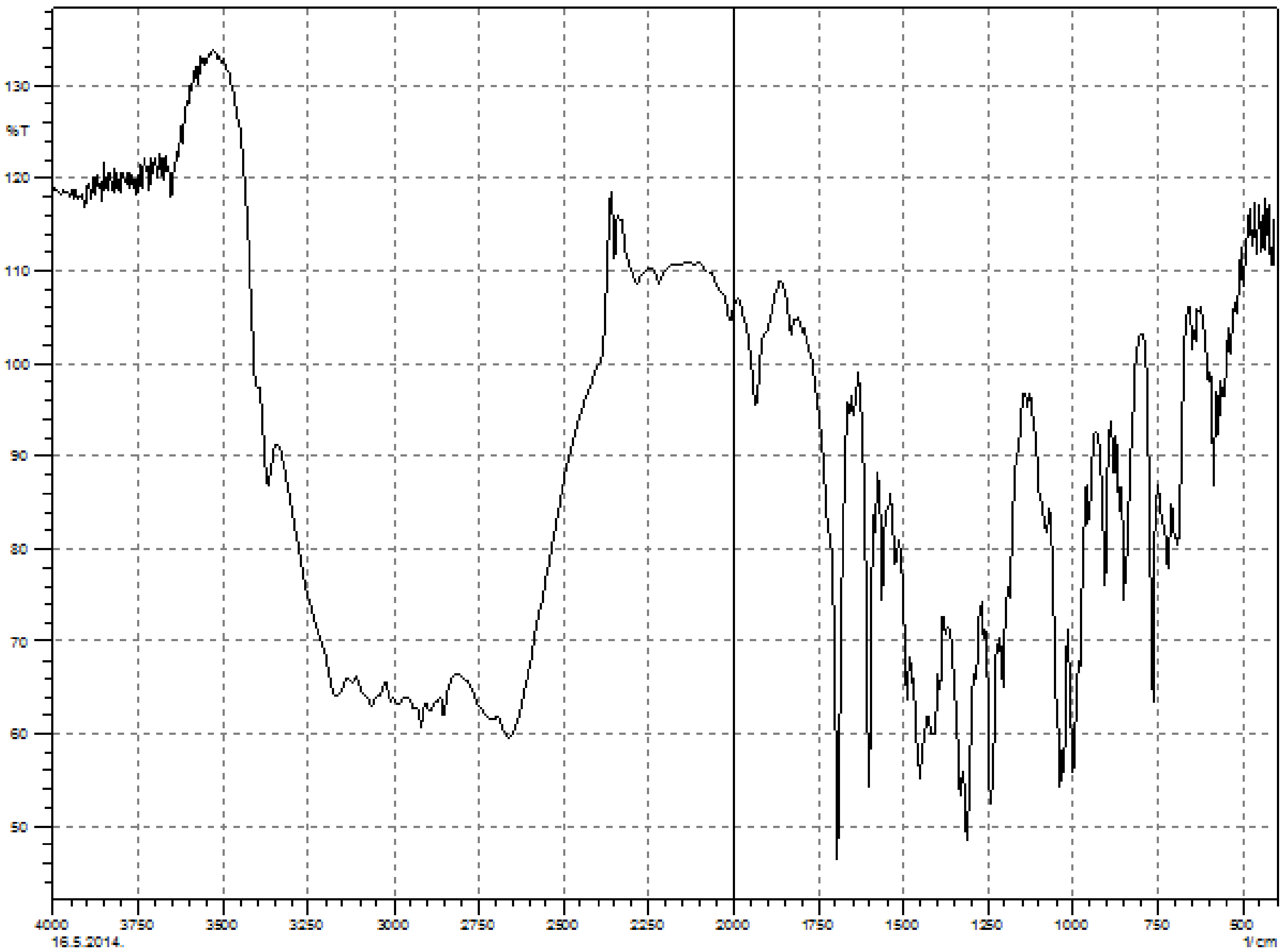

Figure S23. ^{1}H-NMR spectrum of compound **9** in DMSO-d_6 solution.

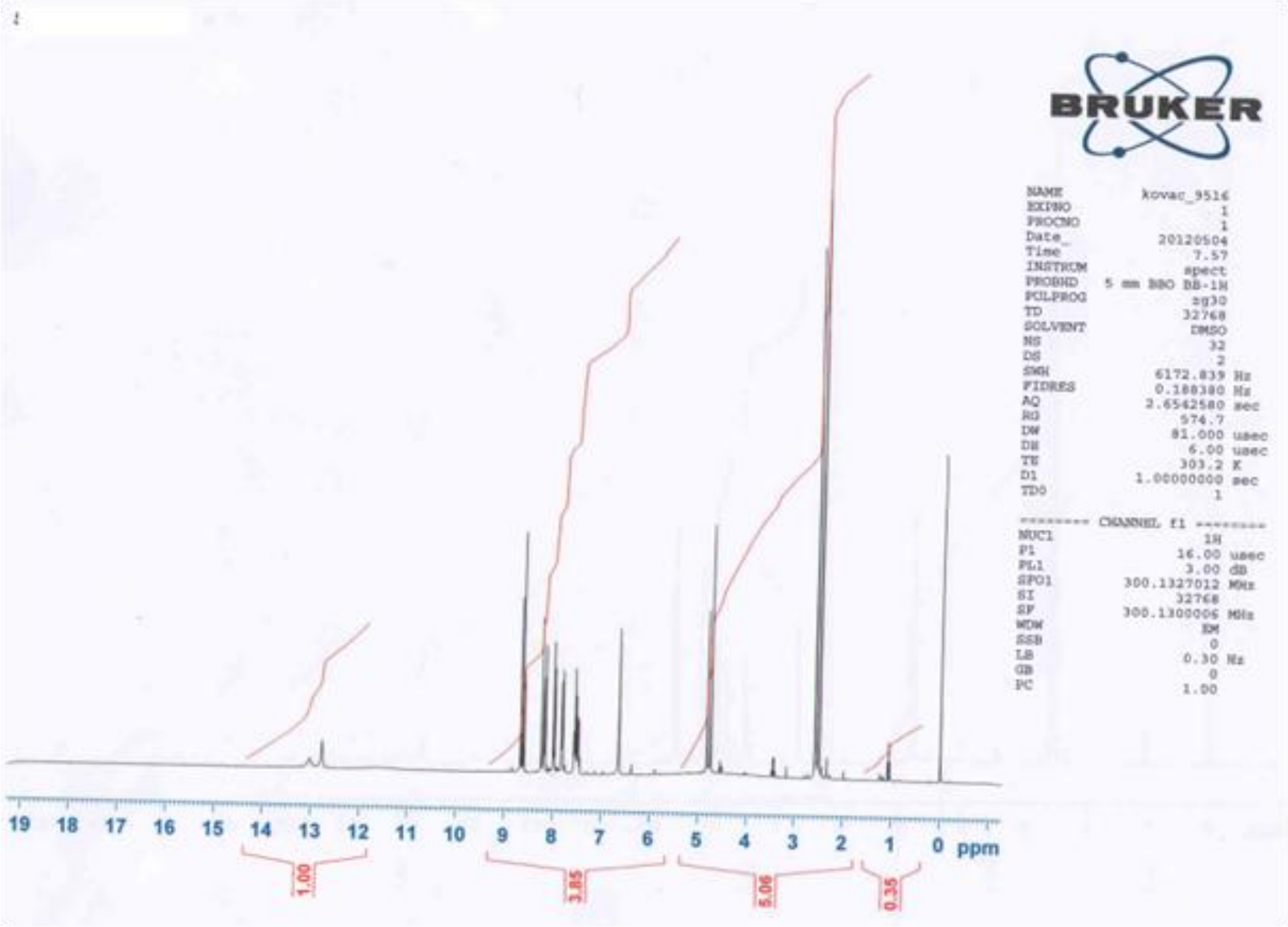

Figure S24. ^{13}C-NMR spectrum of compound **9** in DMSO-d_6 solution.

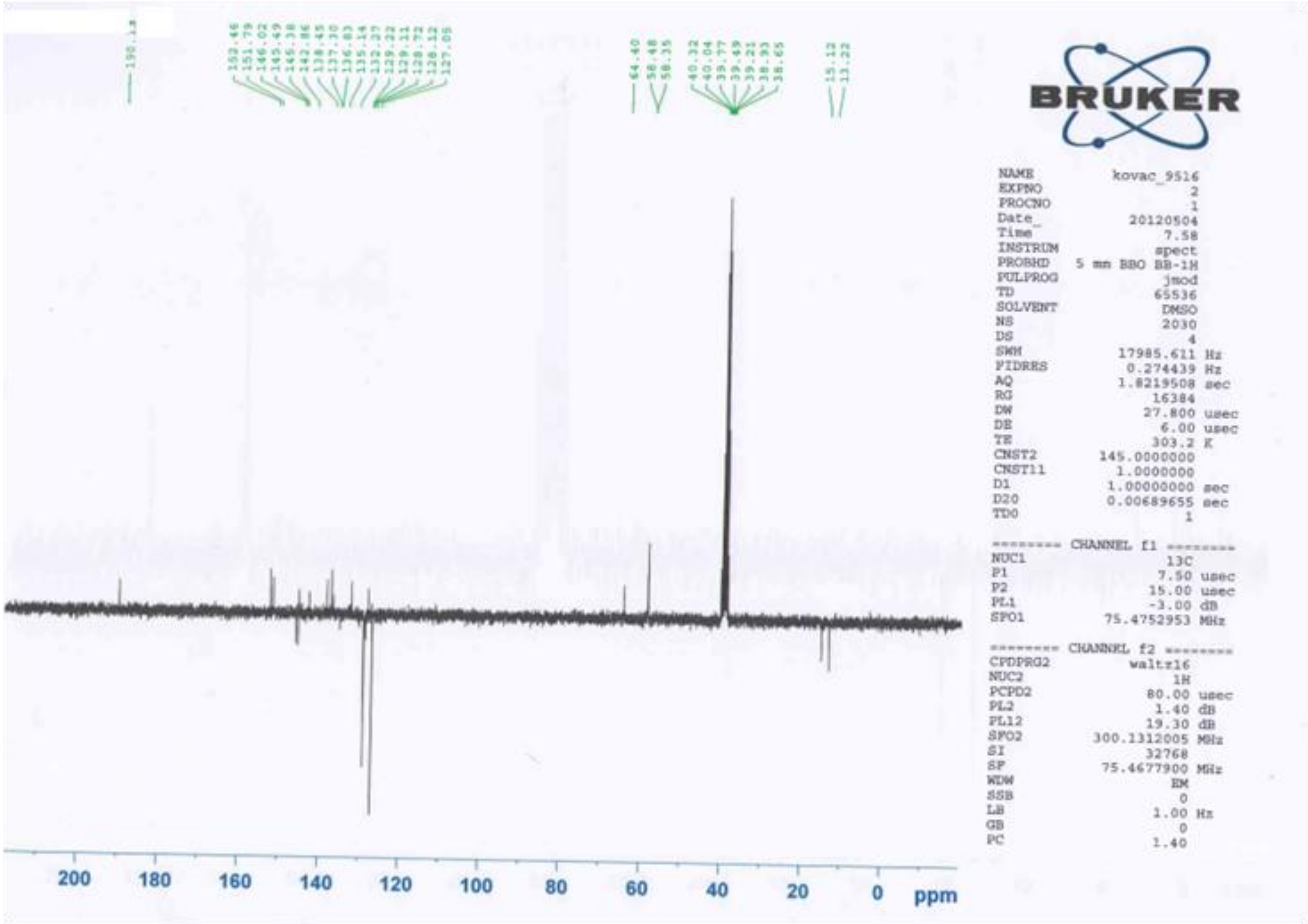

Figure S25. FT-IR spectrum of compound **10.**

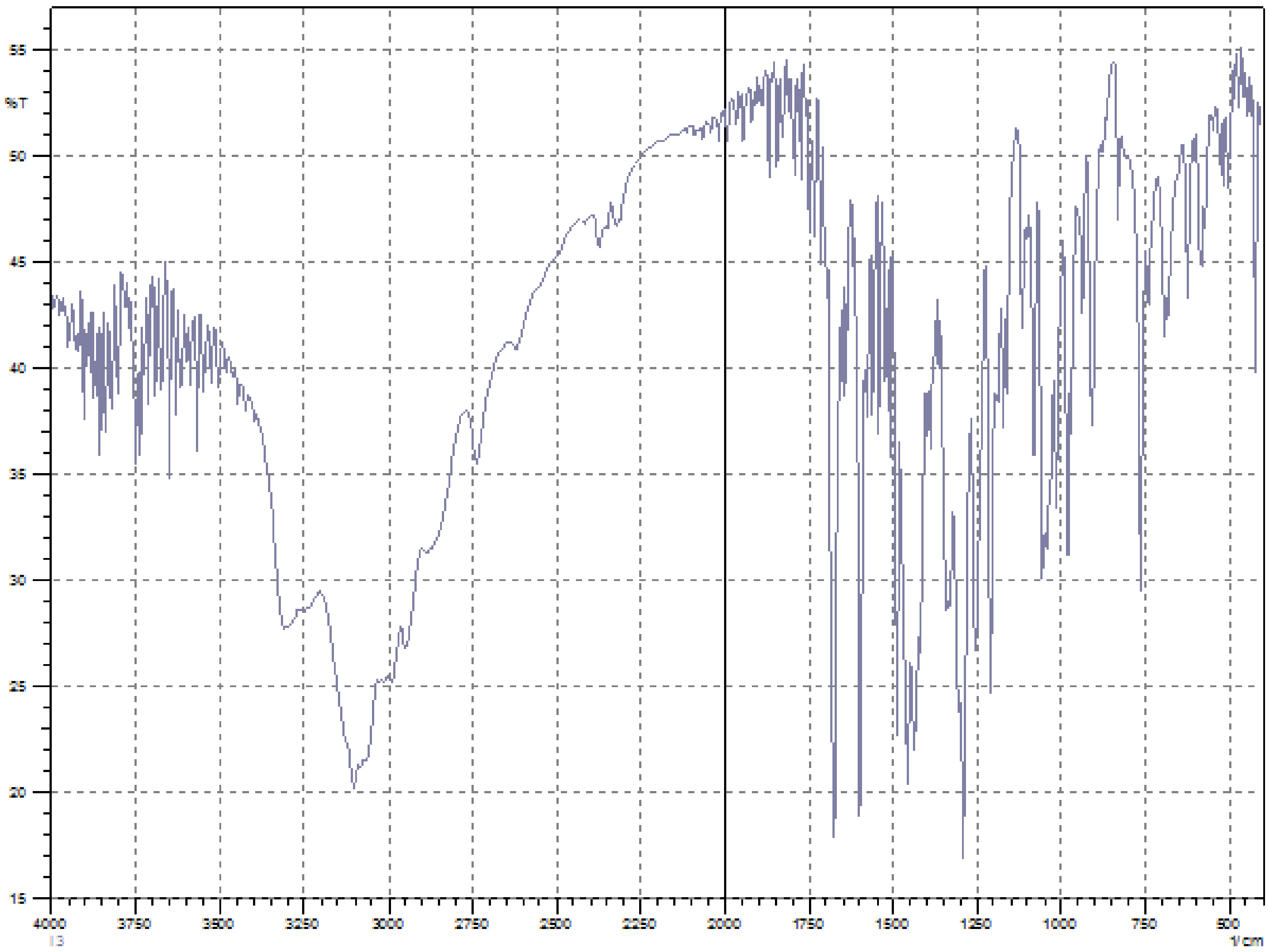

Figure S26. ^{1}H-NMR spectrum of compound **10** in DMSO-d_6 solution.

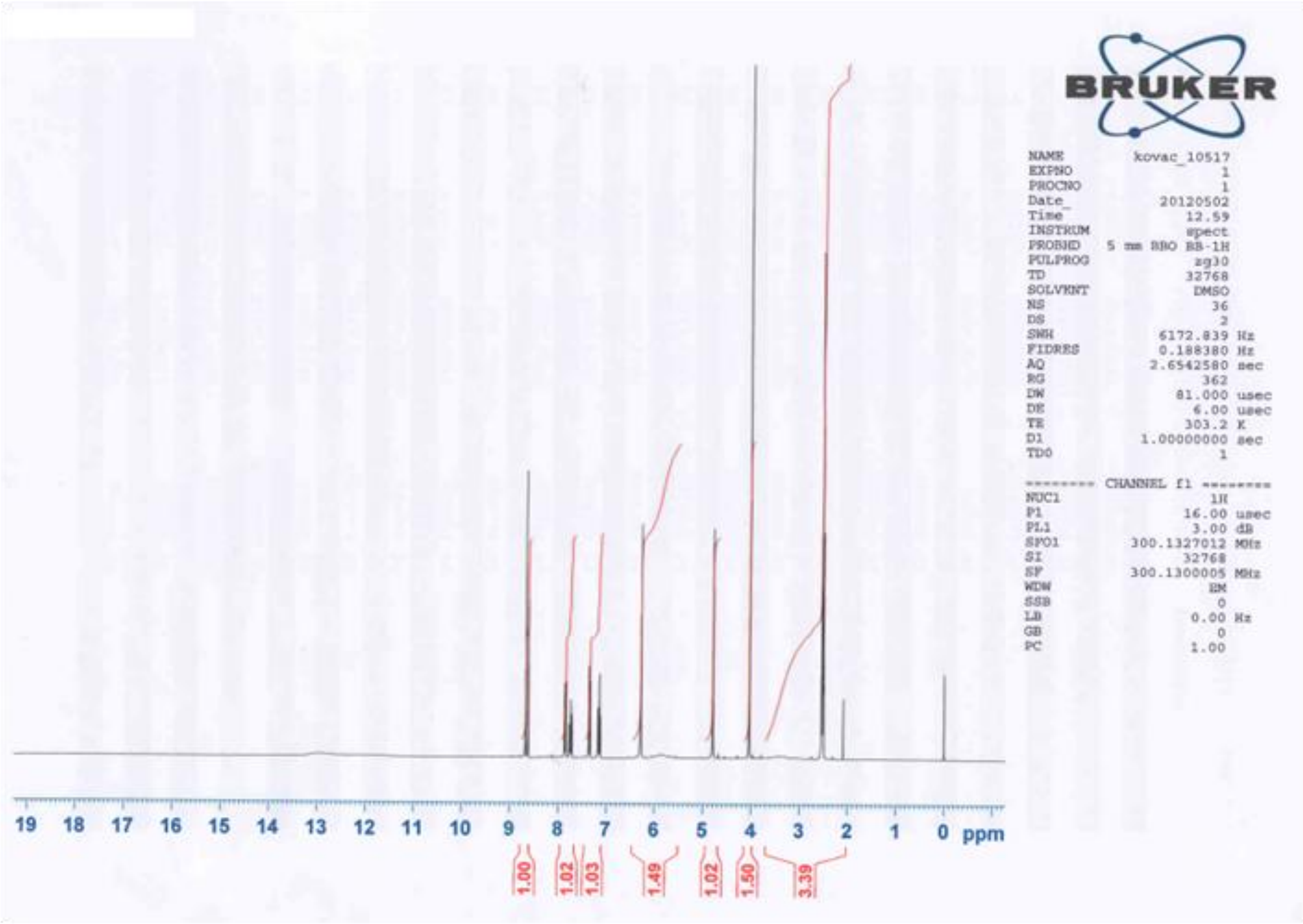

Figure S27. ^{13}C-NMR spectrum of compound **10** in DMSO-d_6 solution.

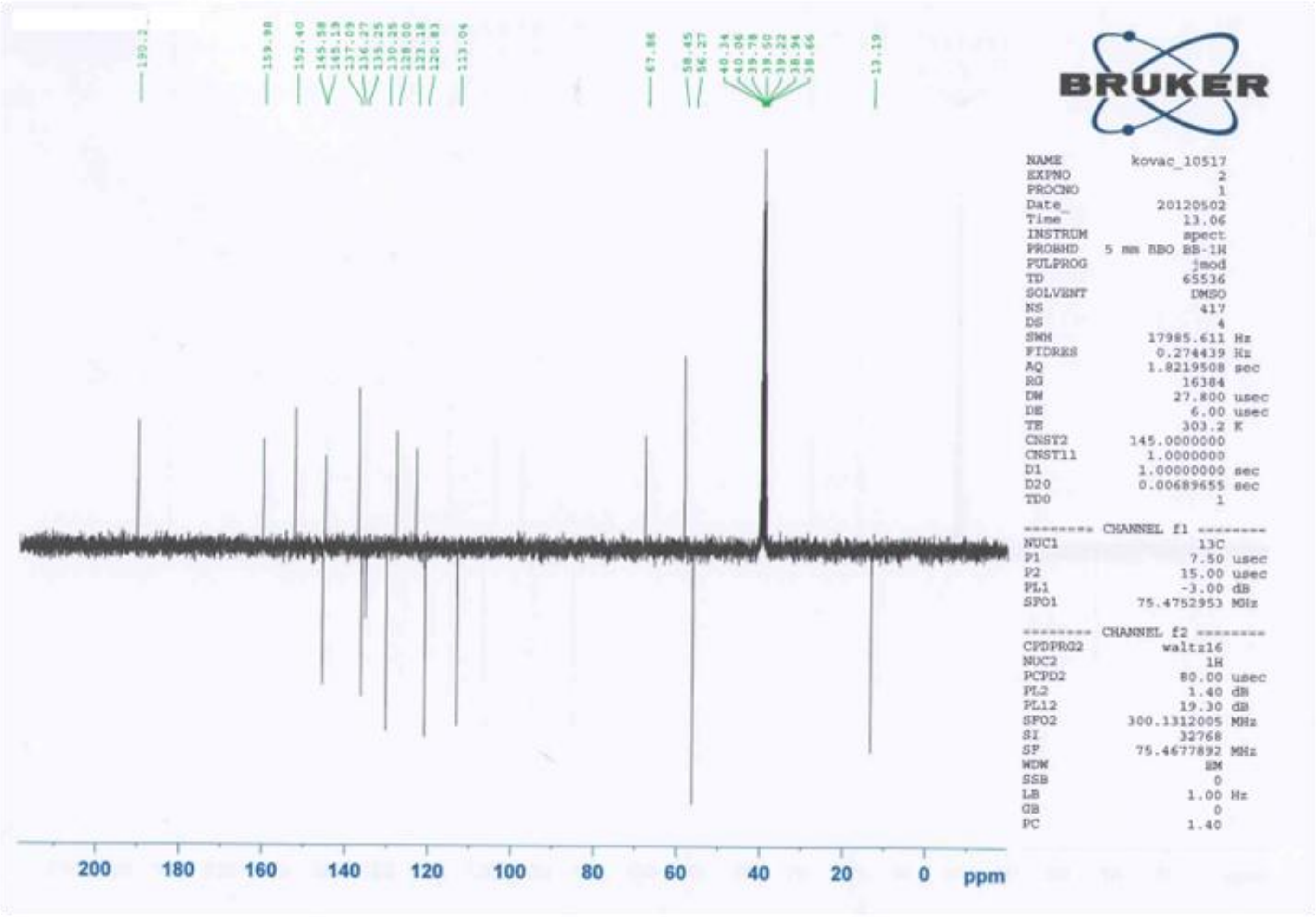

Supplement: Supplementary file 1 [file molecules-19-07610-s001.pdf]
